# Exploring a repurposed candidate with dual hIDO1/hTDO2 inhibitory potential for anticancer efficacy identified through pharmacophore-based virtual screening and in vitro evaluation

**DOI:** 10.1038/s41598-024-59353-4

**Published:** 2024-04-24

**Authors:** Nourhan M. Aboomar, Omar Essam, Afnan Hassan, Ahmad R. Bassiouny, Reem K. Arafa

**Affiliations:** 1 Drug Design and Discovery Lab, Zewail City of Science and Technology, Ahmed Zewail Road, October Gardens, Cairo, 12578 Giza Egypt; 2 Biomedical Sciences Program, University of Science and Technology, Zewail City of Science and Technology, Cairo, 12578 Egypt; 3 Euro-Mediterranean Master in Neuroscience and Biotechnology Program, Alexandria University, Alexandria, 21511 Egypt; 4 Department of Biochemistry, Faculty of Science, Alexandria University, Alexandria, 21511 Egypt

**Keywords:** Human Indoleamine 2,3-dioxygenase 1 (hIDO1), Human Tryptophan 2,3-Dioxygenase 2 (hTDO2), Dual inhibitors, Cell cycle arrest, Pitavastatin, Drug repurposing, Biochemistry, Biological techniques, Cancer, Drug discovery, Chemistry

## Abstract

Discovering effective anti-cancer agents poses a formidable challenge given the limited efficacy of current therapeutic modalities against various cancer types due to intrinsic resistance mechanisms. Cancer immunochemotherapy is an alternative strategy for breast cancer treatment and overcoming cancer resistance. Human Indoleamine 2,3-dioxygenase (hIDO1) and human Tryptophan 2,3-dioxygenase 2 (hTDO2) play pivotal roles in tryptophan metabolism, leading to the generation of kynurenine and other bioactive metabolites. This process facilitates the de novo synthesis of Nicotinamide Dinucleotide (NAD), promoting cancer resistance. This study identified a new dual hIDO1/hTDO2 inhibitor using a drug repurposing strategy of FDA-approved drugs. Herein, we delineate the development of a ligand-based pharmacophore model based on a training set of 12 compounds with reported hIDO1/hTDO2 inhibitory activity. We conducted a pharmacophore search followed by high-throughput virtual screening of 2568 FDA-approved drugs against both enzymes, resulting in ten hits, four of them with high potential of dual inhibitory activity. For further in silico and in vitro biological investigation, the anti-hypercholesterolemic drug Pitavastatin deemed the drug of choice in this study. Molecular dynamics (MD) simulations demonstrated that Pitavastatin forms stable complexes with both hIDO1 and hTDO2 receptors, providing a structural basis for its potential therapeutic efficacy. At nanomolar (nM) concentration, it exhibited remarkable in vitro enzyme inhibitory activity against both examined enzymes. Additionally, Pitavastatin demonstrated potent cytotoxic activity against BT-549, MCF-7, and HepG2 cell lines (IC_50_ = 16.82, 9.52, and 1.84 µM, respectively). Its anticancer activity was primarily due to the induction of G1/S phase arrest as discovered through cell cycle analysis of HepG2 cancer cells. Ultimately, treating HepG2 cancer cells with Pitavastatin affected significant activation of caspase-3 accompanied by down-regulation of cellular apoptotic biomarkers such as IDO, TDO, STAT3, P21, P27, IL-6, and AhR.

## Introduction

Cancer is a significant cause of death globally, second only to cardiovascular disease, with approximately 18.1 million cases and 10 million deaths in 2020. The number of new cases increases steadily each year with expectations to double within the next 30 years^1^. Cancer originates from normal cells that undergo DNA mutations, leading to deformations and uncontrollable cell division. Various genetic and environmental factors contribute to this abnormal behavior^2^. The Kynurenine (Kyn) pathway was identified among pathways involved in cancer progression. In this pathway, the majority, about 95%, of the tryptophan (Trp) present in the human body undergoes metabolism to form Kyn, generating toxic metabolites^3^. These metabolites are implicated in creating an immune-suppressive milieu, fostering tumor development by inducing anergy in effector T cells and promoting the proliferation of regulatory T cells. Additionally, the oncogenic metabolite Kyn activates the Aryl Carbon Receptor (AhR) pathway, which serves as a pro-tumoral factor, influencing the invasive properties of cancer cells^4^. The human Indoleamine 2,3-Dioxygenase 1 (hIDO1) enzyme predominantly catalyzes the conversion of Trp to Kyn, playing a pivotal role in initiating and regulating the Kyn pathway^5^. Targeting hIDO1 has emerged as a prominent strategy in cancer immunotherapy. However, tumor cells have shown the ability to compensate for inhibited hIDO1 activity by upregulating another critical enzyme, human Tryptophan 2,3-dioxygenase 2 (hTDO2)^6^. Recent clinical trials have underscored the limited efficacy of hIDO1 inhibition alone, particularly evident in phase III trials of Epacadostat^7^. Moreover, activation of the Aryl Carbon Receptor (AhR) by hIDO1 inhibitors has been associated with promoting carcinogenic effects in various human cancers, potentially correlating with unfavorable prognosis. Yet, the long-term impact of sustained AhR activation on cancer progression remains uncertain. Notably, hIDO1 inhibitors can mimic Trp as a false nutrient signal, leading to the reactivation of mammalian target of rapamycin (mTOR) activity, which is typically suppressed by Trp depletion. This phenomenon may artificially enhance the antitumor activity of these inhibitors^8^. Consequently, the dual inhibition of hIDO1/hTDO2 emerges as a more promising strategy for effectively targeting the Trp-Kyn pathway. Both hIDO1 and hTDO2 represent pivotal targets in cancer treatment research, offering potential avenues for refining approaches to inhibit the Kyn pathway and combat cancer progression.

Presently, numerous small molecules exhibiting efficacy against either hIDO1 or hTDO2 have been synthesized and progressed to clinical trials. These encompass Epacadostat (phase III), indoximod (phase III), NLG-802 (a prodrug of indoximod) (Phase I), BMS-986205 (Phase III), PF-06840003 (Phase I), navoximod (Phase I), KHK2455 (Phase I), and LY3381916 (Phase I)^9^. Aditionally, only four compounds have been developed as dual inhibitors of hIDO1/hTDO2 and are currently undergoing phase I clinical trials: HTI-1090, LPM-3480226, DN1406131, and M4112. Nevertheless, none of these dual inhibitors have yet received FDA approval.

Given the absence of FDA-approved inhibitors targeting both hIDO1 and hTDO2, repurposing existing drugs presents a viable strategy for exploring the potential efficacy of FDA-approved medications in inhibiting both enzymes for cancer treatment. This study aims to repurpose FDA-approved drugs as prospective dual inhibitors for hIDO1/hTDO2 by designing a ligand-based pharmacophore model tailored to these enzymes. Subsequently, the model is applied in a pharmacophore-based virtual screening of a curated list of FDA-approved drugs against both targets. The best four hits are scrutinized based on their docking scores, adherence to the pharmacophore model, and binding affinity to the respective enzyme pockets, culminating in the selection of “Pitavastatin” as the most promising candidate. Pitavastatin undergoes further evaluation through in silico methods and in vitro cytotoxicity assessments using enzymatic assays (hTDO2 and hIDO1 fluorogenic inhibitor screening assays). The cytotoxic effects of Pitavastatin are examined through an in vitro cytotoxicity assay (MTT-based) across three distinct tumor cell lines (BT-549, MCF-7, and HepG2), with MCF-10A (normal breast epithelial cells) serving as the negative control. Evaluation of Pitavastatin's cytotoxic effect includes flow cytometry and cell cycle analysis on HepG2, the cell line demonstrating the most favorable reported IC_50_. Furthermore, the study investigates the expression of specific genes implicated in the Trp/Kyn pathway using ELISA and RT-PCR. Our findings suggest that Pitavastatin exhibits dual inhibitory effects on the hIDO1/hTDO2 thus hampering the Kyn pathway, indicating its valid potential as an effective agent in anti-cancer therapy.

## Methodology

### Pharmacophore training set preparation, model generation, and validation

A training set of 12 hIDO1/hTDO2 dual inhibitors gathered from previous studies was compiled with emphasis that their reported IC_50_ values be less than 1 µM (Table 1)^10–13^. Using MOE v.2019.01, a ligand-based pharmacophore model was built based on the designed training set where flexible alignment protocol was applied on the training set with an energy cutoff value of 10, iteration limit of 100 conformations, and failure limit of 10 times. Force field charges were calculated via priority search and the conformation search was done by the stochastic method. The pharmacophore features were extracted by calculating the consensus score with a threshold of 50% and a tolerance value of 1.15.

**Table 1:** The training set used for building a pharmacophore model.

Item	Compound	IC_50_ (µM)		Pharmacophore fitting	Fitting score
		**IDO1**	**TDO2**		
**1**		0.005	0.004		4/5
**2**		0.06	0.025		5/5
**3**		0.040	0.034		5/5
**4**		0.045	0.042		5/5
**5**		0.030	0.048		5/5
**6**		0.358	1.029		4/5
**7**		0.70	0.360		4/5
**8**		0.29	0.220		5/5
**9**		1.09	0.085		5/5
**10**		0.008	0.065		4/5
**11**		0.108	0.91		4/5
**12**		0.886	0.858		5/5

For validation of the generated pharmacophore model, three different methods were executed. The first is internal validation by re-screening the training set of compound shown in Table 1 against the pharmacophore model. Subsequently, external validation was performed initially by using a test set of other 56 active compounds obtained from the literature with reported dual inhibitory activity against hIDO1/hTDO2 (IC_50_ ≤ 1 µM) (compounds’ structures are not shown). Finally, another external validation was conducted utilizing a decoy set of 207 compounds (IC_50_ > 20 µM) collected from the literature (compounds’ structures are not shown). The model quality was assessed by calculating the model sensitivity (True Positive Rate (TPR)), specificity, False Positive Rate (FPR), positive and negative predictive values, and accuracy using the following equations:

1 $${\text{Sensitivity}}\; \, \left( {{\text{TPR}}} \right) \, = \frac{Active\; hits}{{Total\; Active \;compounds}},$$

2 $${\text{Specificity }} = \frac{True \; negatives}{{No. \;Decoys}},$$

3 $${\text{False}}\;{\text{ Positive}}\;{\text{ Rate }}\left( {{\text{FPR}}} \right) \, = \frac{False \;positives}{{No. \; Decoys}},$$

4 $${\text{Accuracy }} = \frac{Active\; hits + True \;Negatives}{{Total\; Actives\& Decoys}}$$

5 $${\text{Positive }}\;{\text{predictive}}\;{\text{ value }} = \frac{True \; positives}{{True \; Positives + false \; positives}}$$

6 $${\text{Negative }}\;{\text{Predictive}}\;{\text{ value }} = \frac{True \; Negatives}{{True\; negatives + false\; negatives}}$$

### Pharmacophore filtration of FDA approved drugs dataset

In the pursuit of identifying the optimal candidate, a total of 2568 FDA-approved drugs were obtained from the drug data bank website in the form of an SDF file^14^. Subsequently, this database was subjected to pharmacophore screening employing the designed pharmacophore model. Further refinement of the filtered database was conducted through a manual filtration process based on Lipinski’s and Veber’s rules for drug-like molecules, utilizing the MOE v.2019.01 software. The resultant refined set of candidates was then subjected to virtual screening.

### Protein preparation for docking

**Figure 1:**
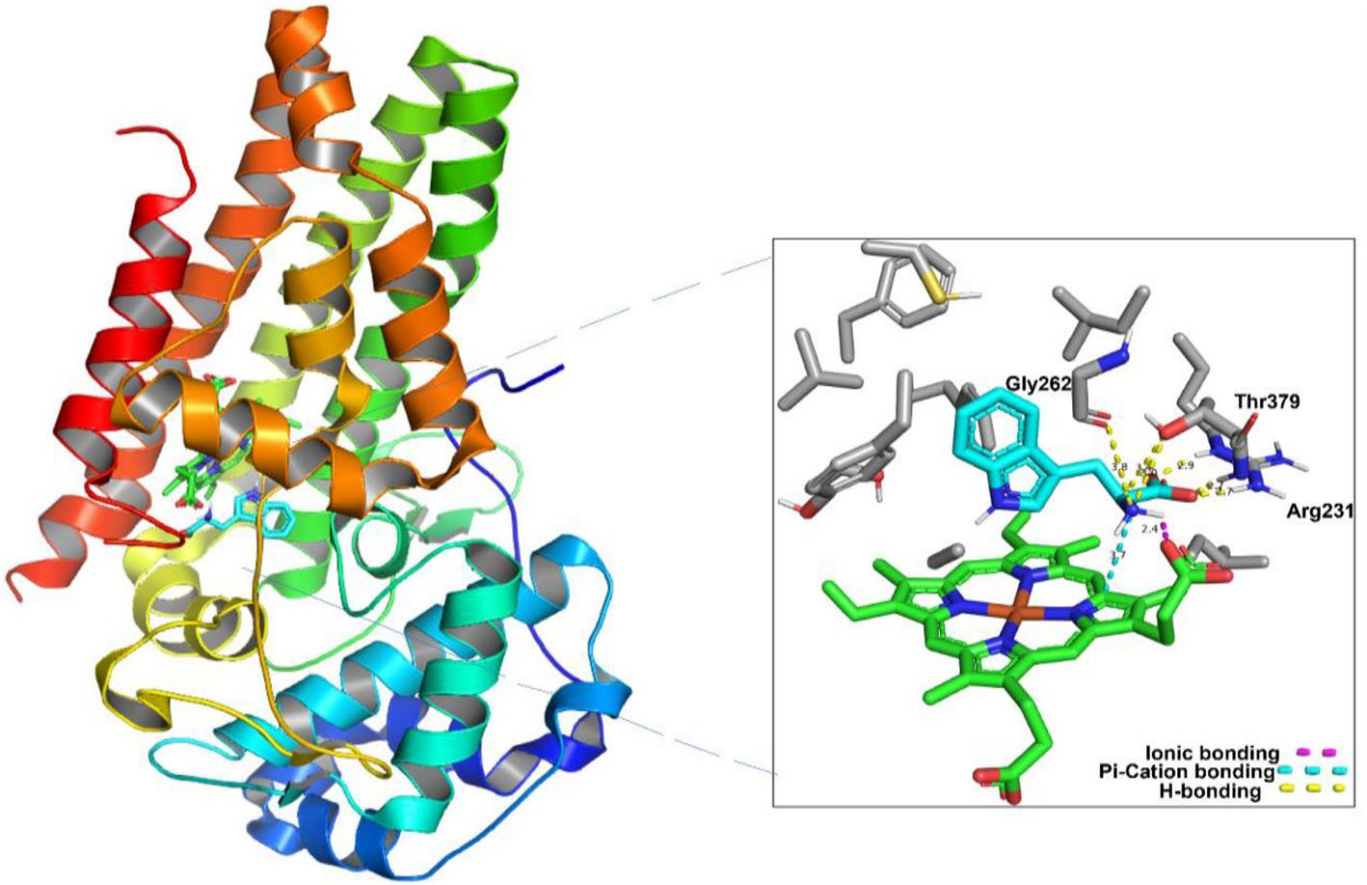
hIDO1 and its heme group (shown in green) in complex with Tryptophan (shown in Cyan) (PBD: 6E46).

**Figure 2:**
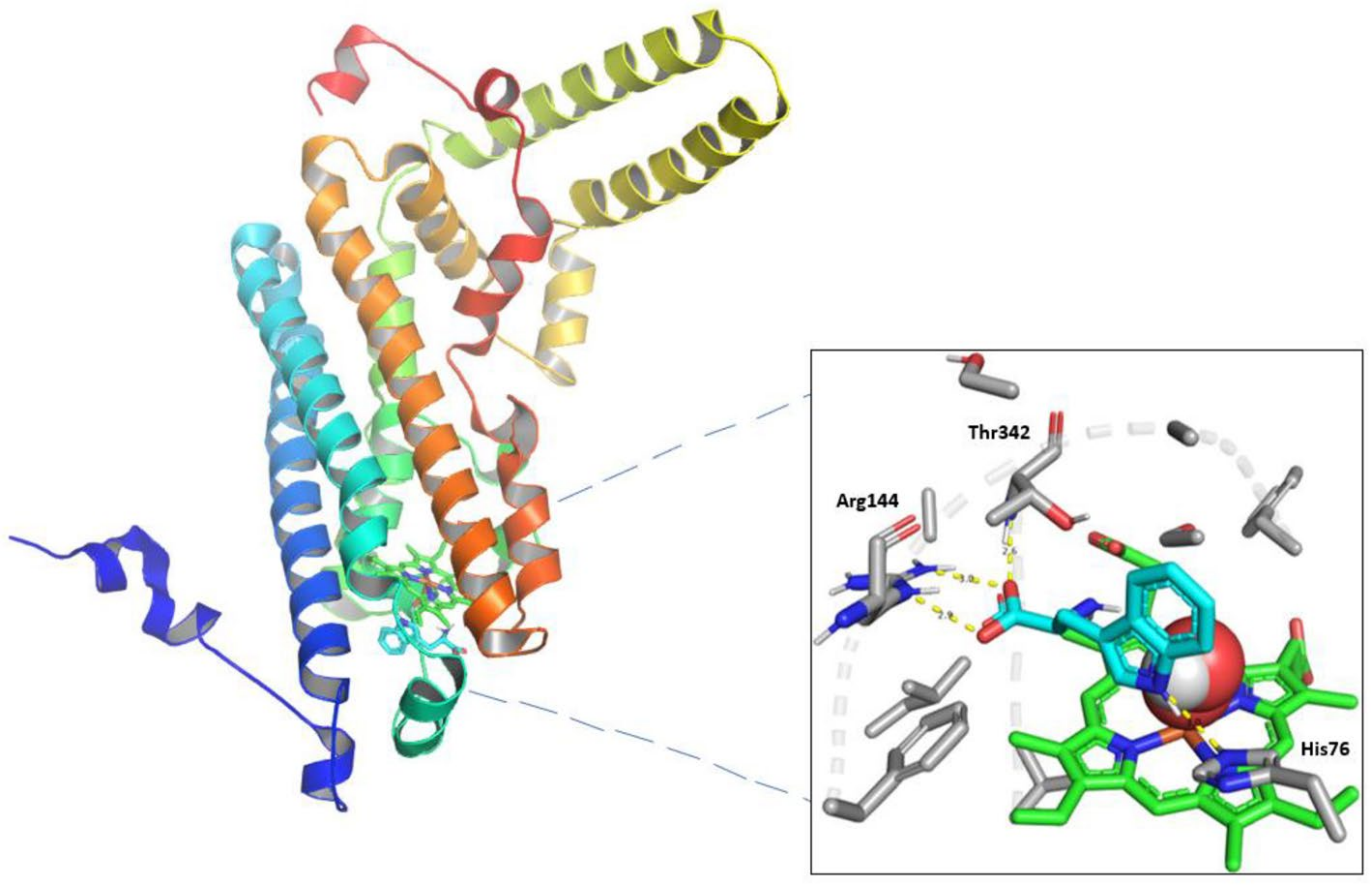
hTDO2 and its heme group (Shown in green) in complex with Tryptophan (shown in Cyan) and Oxygen molecule (Shown as red spheres) (PBD: 5TI9).

The active sites of both enzymes; hIDO1 and hTDO2, were studied via downloading their crystal structures complexed with Tryptophan (Trp) from Protein Data Bank (PDB) (PDB ID. 6E46 and 5TI9 for hIDO1 and hTDO2, respectively). Both enzymes were found to display high structural similarity at the active site, as shown in Figs. 1 and 2. Both active sites are divided into two pockets; A and B perpendicular to each other. The heme group is located at the intersection of the 2 pockets with which Trp forms a coordinate bond^15^. The main difference between both enzymes is that Trp catabolism is performed by Ser167 in hIDO1 via a water-mediated hydrogen bond, while in hTDO2 His76 acts as a base catalyst for Trp metabolism via a direct hydrogen bond^16^. In hIDO1, the carboxylate moiety of Trp forms an ionic bonding with Arg231, which is important for Trp recognition and binding. Moreover, the indole moiety is localized in pocket A, while the carboxylate and ammonium groups extending into B pocket create networks of hydrogen bonds with Gly262, Thr381, conserved GTGG motif located in JK-loop, and a 7-propionate group of the heme. Also, the indole group of the Trp is in line with hydrophobic amino acids such as Leu234, Phe226, Val 130, Phe163, and Tyr126^17^.

On the other hand, eight residues in hTDO2 were reported to have a crucial rule in hTDO2 activity. Arg144 recognizes Trp while Phe72, His76, and Phe140 participate in heme and Trp binding. In addition, Tyr42 and Tyr45, found in the outspread N-terminal domain, take part in Trp binding, while His328 binds with heme in the proximal heme pocket^18^. Finally, Ser151 participates in binding with heme.

Conclusively, the active site of hIDO1 demonstrates higher structural flexibility than that of hTDO2, allowing catalysis of a broad range of substrates. In contrast, hTDO2 is specific for Trp only^19^.

The crystal structure retrieved from the Protein Data Bank for human IDO1 (hIDO1) enzyme was a complex with 1-(4-cyanophenyl)-3-[[3-(2-cyclopropyl ethynyl)imidazo[2,1-b][1,3]thiazol-5-yl]methyl]urea, expressed in *Escherichia coli* BL21(DE3) (PDB ID: 6KPS) with a resolution of 2.25 Å, R-Value Work of 0.204, and R-Value Free of 0.243 (https://www.rcsb.org/structure/6kps). The crystal structure retrieved for human TDO2 (hTDO2) enzyme was in complex with (3S)-3-(5-fluoro-1H-indol-3-yl)pyrrolidine-2,5-dione expressed in *Escherichia coli* (PDB ID: 6PYY) with a resolution of 2.40 Å, R-Value Work of 0.191, and R-Value Free of 0.237) (https://www.rcsb.org/structure/6pyy).

MOE v.2019.01 was used to prepare both crystal structures for virtual screening utilizing Merck Molecular Force Field 94X (MMF94X) as its parameters are suitable for gas phase small molecules in medicinal chemistry providing good accuracy across a range of organic and drug-like molecules. Moreover, it employs the internal bond-charge-increment charge model, and it is compatible with the Born solvation model. The 3D protonation feature provided in MOE v.2019.01 was applied for hydrogen atoms addition besides allowing ASN, GLN, and HIS flips during the protonation process. Finally, water molecules at a distance larger than 4.5 Å from either the receptors or the ligands were deleted, followed by complex refining to an RMS gradient value of 0.1 kcal/mol/Å.

### Re-docking of the co-crystallized ligands and high throughput virtual screening of FDA-approved drugs

Self-docking was performed for the ligands co-crystallized with hIDO1 and chain A of hTDO2 (PDB IDs: 6KPS and 6PYY, respectively) via MOE v.2019.01 for the sake of validating the docking protocol using the induced fit protocol and triangle matcher method. dG was identified as the first rescoring function while GBVI/WSA dG was adopted as a second rescoring function. Finally, refinement was implemented employing MMF94x force field. Similarly, virtual screening of a filtered test set (308 FDA-approved drugs) was performed against the active sites of hIDO1 and chain A of hTDO2 employing the same parameters as those of the docking validation. The top four hits were re-docked against both enzymes using Amber10:EHT force field as (ff10) where Amber10 can parametrize proteins (ff10) while EHT preliminary release parametrizes small molecules. Eventually, the ligands interactions and pocket surface occupancy in both proteins were visualized using PyMOL(TM) 2.5.2 and MOE v.2019.01^20,21^.

### Molecular Dynamics simulations

A Molecular Dynamics (MD) simulation lasting 100 ns was conducted to examine the binding of Pitavastatin to both hIDO1 and hTDO2 receptors, aiming to assess the stability of drug binding within the active domains of these proteins and to gain insights into their interactions. To validate the stability of Pitavastatin, MD simulations of 100 ns were performed for the ligands co-crystallized with hIDO1 and chain A of hTDO2 enzymes (PDB IDs: 6KPS and 6PYY, respectively). The MD simulations were executed using GROMACS-2023.1, employing the AMBER99SB force field for protein topology preparation, and the ACPYPE (or AnteChamber PYthon Parser interfacE) server for ligand topology preparation. During the solvation process, a dodecahedral unit cell box was utilized, along with periodic boundary conditions set at 10 Å. Ions were incorporated using the steepest descent minimization algorithm, with sodium and chloride ions used for protein neutralization. Energy minimization was employed to mitigate steric clashes in the complex, employing the steepest descent minimization algorithm, with a force cutoff set at 10.0 kJ/mol, and a maximum of 50,000 steps. Subsequently, two equilibration processes were conducted: NVT and NPT equilibration, employing a modified Berendsen thermostat and leap-frog integrator for 50,000 steps, equivalent to 10 picoseconds. Finally, the MD simulation was run for 100 ns with a time step of 2 femtoseconds for each step.

### Pitavastatin extraction and purification

Pitavastatin was chosen to be the best hit for further in vitro investigation as it fulfills all the 5 features of the designed ligand-based pharmacophore model for hIDO1/hTDO2 dual inhibition with very good binding affinities to both enzymes and interacting with the main amino acids in both enzymes’ pockets mimicking the native ligand Trp. Accordingly, Pitavastatin calcium was obtained through extraction and purification from Lipidalon tablets 4 mg from (Mash Premiere for Pharmaceutical Industries, Egypt). First, the tablets were washed with distilled water to remove the yellow coat then dried and ground into powder. Solid-liquid extraction with chloroform was performed to extract Pitavastatin. Finally, the organic solvent was evaporated leaving a white solid powder of Pitavastatin calcium. It was purified by dissolving the impurities in a solution of n-hexanes and ethyl acetate. For validation purposes, the FT-IR and melting point were measured and compared with the corresponding reported data for Pitavastatin.

### In vitro hIDO1 and hTDO inhibition screening assay

IDO1 Inhibitor Screening Assay Kit (Catalog # 72021) and TDO2 Fluorogenic Inhibitor Screening Assay Kit (Catalog # 72039) were used for in vitro enzymatic inhibition assays where serial logarithmic dilutions (concentration from 0.01 to 100 µM) were prepared for Pitavastatin in addition to the positive control reference drugs. Experimental procedures were followed as instructed by the kits’ manufacturer. IC_50_ values of the compounds were calculated from the obtained dose response curves.

### MTT cytotoxicity assay

To determine the toxic effect of Pitavastatin in vitro, MTT cytotoxicity assay was performed on 3 cancer cell lines; tumoral HepG2, BT-549 and MCF-7 in addition to normal breast cell line (MCF-10A) as a negative control in this study. Cell Lines were obtained from American Type Culture Collection (ATCC) and cultured using DMEM (Invitrogen/Life Technologies) supplemented with 10% FBS (Hyclone,), 10 ug/mL of insulin (Sigma), and 1% penicillin–streptomycin*. *In vitro toxicology assay kit (MTT based) was purchased from Sigma where 4 cell lines were exposed to 100 µM, 25 µM, 6.3 µM, 1.6 µM, and 0.4 µM of Pitavastatin and Staurosporine (as a standard) in a 96-cluster well culture plate for 24 h. Cell viability after exposure to Pitavastatin was measured spectrophotometrically measuring the absorbance at a wavelength of 540 nm.

### Flow cytometry apoptosis assay

Apoptosis assay was conducted employing Annexin V-FITC/PI Apoptosis Detection Kit, Cell Signaling Technology (CST) as per the manufacturer’s instructions. HepG2 cancer cells were treated with the pre-determined IC_50_ value of Pitavastatin (1.84 µM) for 48 h. Thereafter, cells were trypsinized and subjected to two PBS-wash steps. Briefly, cells were re-suspended in 5 μL of Annexin V-FITC and 5 μL of PI (staining solution). Then, 0.5 mL of binding buffer was added and mixed with the cells’ suspension. Cells were then incubated in a dark room at 25 °C for only 15 min. Lastly, cells were injected into Cytek® Northern Lights 2000 spectral flow cytometer (Cytek Biosciences) and SpectroFlo™ Software version 2.2.0.3 (Cytek Biosciences, USA) was utilized for quadrant analysis.

### Cell cycle analysis

HepG2 cells were treated with the pre-determineded IC_50_ values of Pitavastatin for 48 h. Subsequently, cells were trypsinized and washed twice with PBS. This was followed by fixation in ice-cold 60% ethanol and another wash step with PBS. Then, 500 µL of propidium iodide (PI) together with RNase staining buffer from Cell Signaling Technology (CST) were added to re-suspend the cells. The cells were then left for 15 min of incubation with the stain. At the end, cells were injected into a Cytek® Northern Lights 2000 spectral flow cytometer (Cytek Biosciences). An estimate of 10,000 cells were obtained from each sample. Analysis of cell population in each phase of the cell cycle was performed using SpectroFlo™ Software version 2.2.0.3 (Cytek Biosciences).

### ELISA assay

HepG2, the most affected cancer cell line upon Pitavastatin treatment, was utilized to evaluate the expression levels of hIDO1, hTDO2, STAT3, P21, P27 and IL-6 using Sigma enzyme linked immunosorbent assay (ELISA) Kit. ELISA assay applies a quantitative sandwich immunoassay using a monoclonal antibody pre-coated microtiter plate wells with a biotin-conjugated polyclonal antibody.

### RNA extraction and quantitative real-time reverse transcription-PCR (RT-PCR)

iScript™ One-Step RT-PCR Kit with SYBR® Green (Bio-Rad Inc., CA, USA) was used for total RNA extraction from HepG2 after treatment with the pre-determined IC_50_ values of Pitavastatin. Total RNA was isolated from the HepG2 cells using the RNAqueous-4PCR kit (Ambion Applied Biosystems, Austin, TX, USA) and cDNA was amplified using 1 μL × 50 iScript Reverse Transcriptase. Quantitative real-time reverse transcription-PCR (RT-PCR) analysis was performed using GAPDH as a housekeeping gene and specific primers that amplify AHR and Caspase-3 genes. The used primers sequences are as follows:

AHR: F 5′- GTCGTCTAAGGTGTCTGCTGGA-3′,

AHR: R 5′- CGCAAACAAAGCCAACTGAGGTG-3′.

Casp3: F 5′- GGAAGCGAATCAATGGACTCTGG-3′,

Casp3: R 5′- GCATCGACATCTGTACCAGACC-3′.

GAPDH: F 5′- GTCTCCTCTGACTTCAACAGCG-3′

GAPDH: R 5′- ACCACCCTGTTGCTGTAGCCAA-3′

### Statistical methods

All results were presented as mean values with their respective standard deviations to facilitate comparison between the control and treatment groups. The statistical significance was determined using an independent sample t-test in SPSS 16.0 software, where a p-value of less than 0.05 denoted a significant difference. Individual comparisons were conducted using Duncan’s multiple range test (SPSS Corp, Chicago, IL). Values were categorized as statistically significant, highly statistically significant, or very highly statistically significant when p-values were less than 0.05, 0.01, or 0.001, respectively.

## Results and discussion

### Pharmacophore modeling and validation

**Figure 3:**
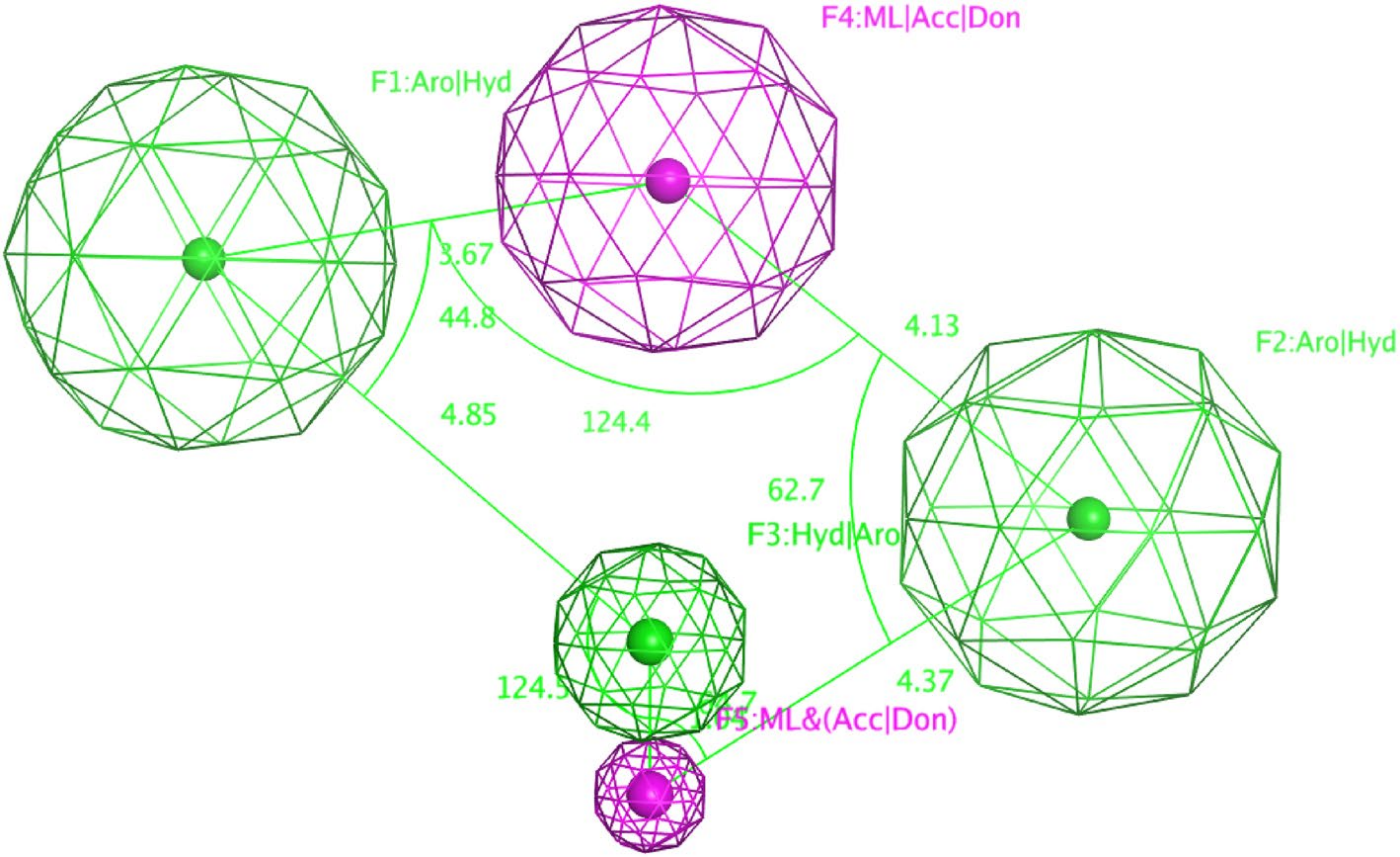
Ligand-based pharmacophore model features.

Five features pharmacophore model (Fig. 3) was generated by applying partial match search on (at least four features) a training set of 12 dual hIDO1/hTDO2 inhibitors. The five features of the model are F1:Aro|Hyd (Radius = 1.5 Å), F2:Aro|Hyd (Radius = 1.5 Å), F3:Aro|Hyd (Radius = 0.7 Å), F4:ML|Acc|Don (Radius = 1.3 Å) and F5:ML&(Acc|Don) (Radius = 0.4 Å); where Aro|Hyd is the aromatic or hydrophobic center, Acc. is H-bond acceptor and Don is H-bond donor. Only four of them are essential whereas F4:ML|Acc|Don is the non-essential feature.

For the two-dimensional properties of the features, the centroid of F1 created an angle of 44.8° with F3 and F4 with distances equal to 4.85 Å and 3.67 Å, respectively. F2 centroid showed an angle of 62.7° between F4 and F5 with distances of 4.13 Å and 4.37 Å, respectively. Moreover, F3 lies between F1, as mentioned earlier, and F5, whose centroid is 1.04 Å away from F3 centroid with an angle of 124.5°. In addition, F4 is located between F1 and F2, as mentioned earlier, with an angle of 124.4°. Finally, F5 is located between F2 and F3 with an angle of 64.7°.

On the other hand, to describe the pharmacophore model in a three-dimensional mode, the pharmacophore XYZ coordinates for each feature was determined: (F1: (0.6502, 2.0583, 2.4823), F2: (− 4.1278, − 0.8281, − 1.5894), F3: (− 0.6952, − 2.0007, 0.1855), F4: (− 0.636, 1.2624, − 0.8650), and F5: (− 0.9913, − 2.8787, 0.6632).

For model validation, internal validation of the training set was performed resulting in identifying all the hits 12 used for building the pharmacophoric model (Table 1). Seven compounds out of the 12 fit all the five pharmacophoric features while 5 compounds fit the four essential features.

Additionally, two types of external validations were conducted to extensively test the model validity before its deployment in the virtual screening process. Initially, a test set of 56 active compounds was used to validate the designed model (compound structures are not shown). This was followed by testing the model with a 207 decoys test set. In both cases the model demonstrated efficiency and reliability. 45 true positives out of 56 active compounds were obtained while only 16 false positives out of 207 decoys were identified. Overall, the model demonstrated a high accuracy of 89.73%, a sensitivity (TPR) of 80.36%, a specificity of 92.27%, a False Positive Rate (FPR) of 7.73%, a positive predictive value of 73.77%, and a negative predictive value of 94.55%.

### Pharmacophore filtration of FDA-approved drugs dataset

Applying pharmacophore search on a dataset of 2568 FDA-approved drugs has resulted in identifying 958 candidates fitting the designed pharmacophore model features. After a second round of filtration through applying Lipinski’s and Veber’s rules of drug-likeness, the number of candidates declined to only 308 candidates. The top 10 FDA-approved drugs were found to be fitting all five or at least the four essential pharmacophore features and are listed in Table 2.

**Table 2:** Pharmacophore model fitting of the best 10 FDA-approved drugs, retained from the virtual screening.

Drug name	Chemical structure	Pharmacophore model fitting	Fitting score
**Trovafloxacin**			5/5
**Vilazodone**			4/5
**Pitavastatin**			4/5
**Dasabuvir**			4/5
**Ranolazine**			4/5
**Acetophenazine**			4/5
**Dasatinib**			4/5
**Gefitinib**			4/5
**Apixaban**			4/5
**Nefazodone**			4/5

### Docking validation and high throughput virtual screening

The co-crystal structures of hIDO1 in complex with 1-(4-cyanophenyl)-3-[[3-(2-cyclopropylethynyl)imidazo[2,1-b][1,3]thiazol-5-yl]methyl]urea (PDB ID: 6KPS) and hTDO2 in complex with (3S)-3-(5-fluoro-1H-indol-3-yl)pyrrolidine-2,5-dione (PDB ID: 6PYY) were obtained from the Protein Data Bank. hIDO1 is a monomer α-helical protein comprising 403 residues and a heme prosthetic group. The large C-terminal domain containing the active site consists of 13 α helices and two 310 helices while the small N-terminal domain contains six-α helices, two short β-sheets, and three 310 helices^22^.

On the other hand, hTDO2 is a homotetramer consisting of 406 residues (15 α-helices without β-strands) and a heme prosthetic group per monomer^23^.

To validate the docking protocol, self-docking of the co-crystallized ligand was performed. The co-crystallized ligands showed poses and interactions identical to the reported ones; RMSD = 1.5325 and 0.3952 Å, S-scores = − 7.96 and − 7.07 kcal/mol, for hIDO1 and hTDO2, respectively.

A virtual screening study was performed on a dataset of 308 FDA-approved drugs; obtained from pharmacophore search and filtration based on Lipinski’s and Veber’s rules, against both hIDO1 and hTDO2 proteins. Regarding hIDO1, the virtual screening results showed that 17 FDA-approved drugs out of 308 drugs were found to have higher S-scores than that of the co-crystallized ligand of PDB ID 6KPS (S = − 7.96 kcal/mol). While in hTDO2 screening, 126 drugs out of 308 drugs reported S-scores better than that of the co-crystallized ligand of PDB ID 6PYY (S = − 7.07 kcal/mol). Therefore, sequential exclusion criteria were implemented for the top 10 compounds based on the formation of interactions with the heme group and the essential residues for inhibition in IDO1 and TDO2 (Arg231 or Ser167 in hIDO1, Arg144 and His76 in hTDO2), the strength of interactions, addressing potential side effects, S scores, and the commercial availability.

The top ten pharmacophore filtered drugs were found to be amongst the compounds displaying good interactions with both hIDO1 and hTDO2 and eliciting good S scores as shown in Table 3. Yet, only 4 drugs out of the 10 formed interactions with the essential residues needed for the inhibition of both enzymes with high capability of fitting into their active sites’ pockets (details in the supplementary material Figs. S1 and S2). Therefore, those four drugs, Trovafloxacin, Vilazodone, Pitavastatin and Dasabuvir, were re-docked and their strength of interactions, S scores, potential side effects of each drug, and the commercial availability were scrutinized. Considering the potential side effects of the four drugs, Trovafloxacin was excluded as it is withdrawn from the market. Also, Vilazodone was excluded due to its potential CNS side effects, while Dasabuvir wasn’t the one of choice by virtue of its potential side effects as a chemotherapeutic agent as well as its commercial unavailability. Amongst the four, Pitavastatin possesses the safest side effects, and demonstrated essential interactions with the active site amino acids of both target enzymes.

**Table 3:** Docking S-scores of the native ligands (self-docking) and the best ten hits docked from the filtered Drug data bank database against hIDO1 and hTDO2.

Item	Drug name	S Score (Kcal/mol)		Reason for exclusion	Pharmacological class
		hIDO1	hTDO2		
1	**Native_1: (**1-(4-cyanophenyl)-3-[[3-(2-cyclopropylethynyl) imidazo[2,1-b][1,3]thiazol-5-yl]methyl]urea)	− 7.96	N/A	N/A	NA
2	**Native_2: (**(3S)-3-(5-fluoro-1H-indol-3-yl) pyrrolidine-2,5-dione)	N/A	− 7.07	N/A	NA
3	**Trovafloxacin**	− 5.32	− 7.36	Withdrawn	Fourth-generation fluoroquinolone antibiotic
4	**Vilazodone**	− 9.04	− 8.57	CNS side effects	Antidepressant serotonin partial agonist reuptake inhibitor (SPARI)
5	**Pitavastatin**	− 7.54	− 6.38	Passed	Antilipemic inhibiting HMG-CoA reductase
6	**Dasabuvir**	− 8.17	− 8.35	Potential side effects	Direct Acting Antiviral (DAA) non-nucleoside NS5B inhibitor
7	**Ranolazine**	− 7.32	− 7.71	Lack of Essential interactions in both enzymes	Second-line antianginal in stable coronary artery disease
8	**Acetophenazine**	− 8.19	− 7.91	Lack of Essential interactions in hTDO2	Antipsychotic postsynaptic D1 and D2 receptors blocker
9	**Dasatinib**	− 7.02	− 8.40	Lack of Essential interactions in both enzymes	Anticancer BCR- ABL kinase inhibitor for management of CML and ALL
10	**Gefitinib**	− 7.34	− 7.53	Lack of Essential interactions in both enzymes	Anticancer EGFR selective inhibitor
11	**Apixaban**	− 8.40	− 8.57	Lack of Essential interactions in hTDO2	anticoagulant reversible direct inhibitor of factor Xa
12	**Nefazodone**	− 8.71	− 8.06	Lack of Essential interactions in both enzymes	Antidepressent Serotonin antagonist and reuptake inhibitor (SARI)

**Figure 4:**
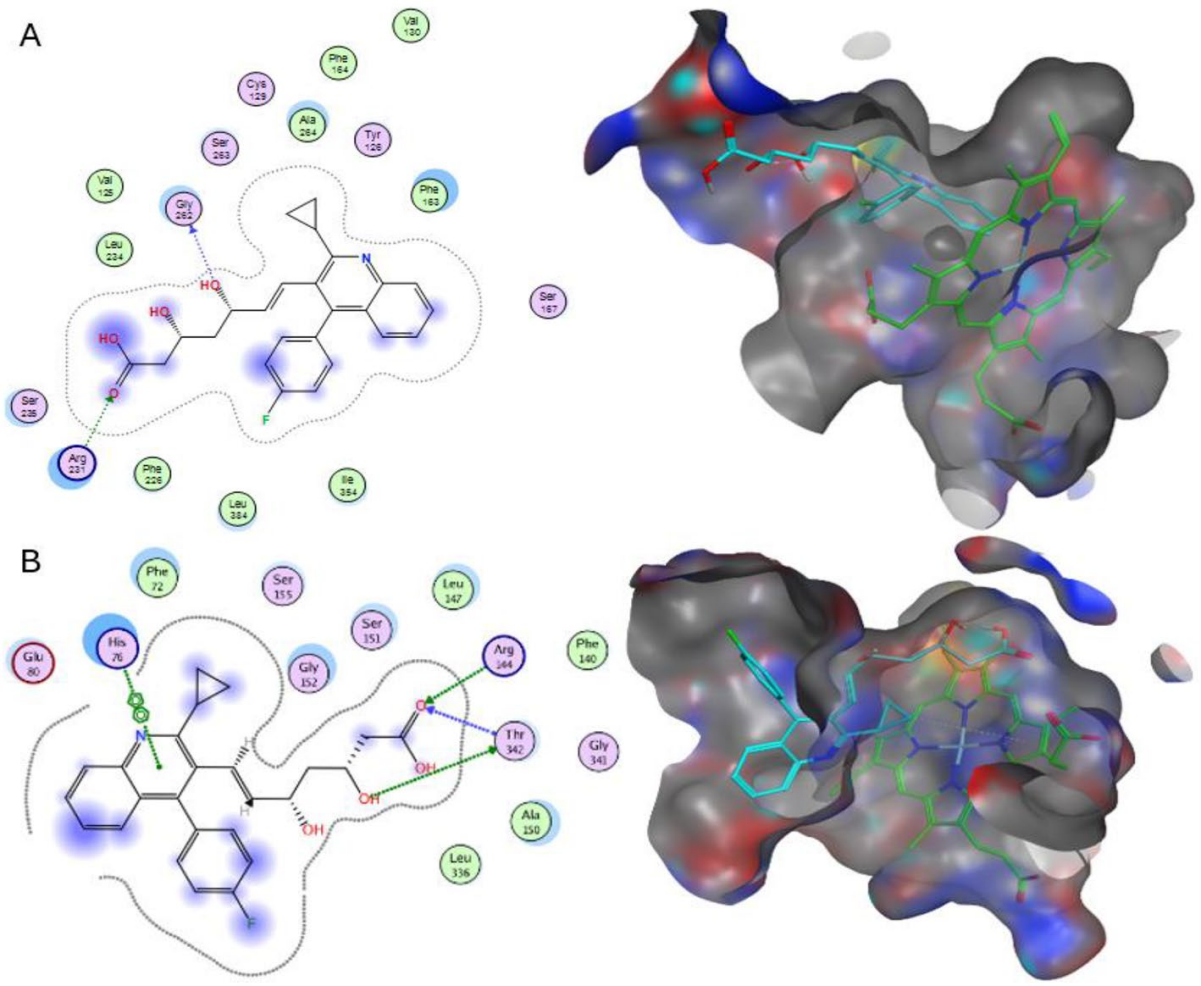
2D interactions and pocket surface occupancy of Pitavastatin, the best hit shown in Cyan, against hIDO1 (**A**) and hTDO2 (**B**). The Heme group is represented in green, the surface map is the Pocket surface occupancy, in which the blue color represents cationic moieties, while red color represents anionic moieties, and the hydrophobic moieties are illustrated in gray.

Analyzing Pitavastatin interactions in both enzymes is shown in Fig. 4. Two non-covalent interactions were visualized in hIDO1 (Fig. 4A). The carboxylic moiety in Pitavastatin could form one hydrogen bond acceptor interaction with Arg231 sidechain while the allylic hydroxyl has a Hydrogen bond donor interaction with the C-terminus of Gly262 backbone in hIDO1. This is highly consistent with previous studies that reported the essentiality of inhibiting Arg231 to prevent Trp coordination to the heme, accordingly, blocking Trp catabolism^24^. Moreover, comparing Fig. 4A to Fig. 1, the interaction with Gly262 indicates the ability of Pitavastatin to occupy pockets A and B of the active site, therefore enhancing the binding affinity to hIDO1 active site.

For hTDO2, as illustrated in Fig. 4B, the quinoline moiety in Pitavastatin formed a π–π interaction with His76 sidechain. Also, the carboxylic acid moiety in Pitavastatin functioned as a Hydrogen bond acceptor with Arg144 sidechain and the N-terminus of Thr342 backbone while the β-hydroxyl moiety interacted with Thr342 sidechain as a Hydrogen bond donor. Finally, the cyclopropyl moiety could form an H-Arene interaction with the heme group, which is consistent with the pharmacophore fitting highlighting cyclopropyl group as an essential hydrophobic feature. The results are consistent with the previous studies reporting the His76 crucial role as a base catalyst responsible for Trp catabolism, in addition to Arg144’s importance in Trp recognition^19^. Also, comparing these interactions to those with Trp in Fig. 2, Pitavastatin could fulfill all the essential interactions needed for hTDO2 inhibition. The β-hydroxy acid moiety in Pitavastatin could mimic the interactions of the Trp amino acid moiety with both Thr342 and Arg144. In addition, the presence of quinoline moiety allowed mimicking the Trp indole group interaction as introducing a larger heteroaromatic core enhances the proximity towards His76 and provides larger occupancy into the active site pocket.

Overall, the interacting moieties in Pitavastatin in both enzymes is consistent with its pharmacophore fitting shown in Table 2.

Pitavastatin could pass all the criteria set for identifying a good dual hIDO1/hTDO2 inhibitor as it could fulfill the essential four features of the ligand-based pharmacophore model generated from a set of dual hIDO1/hTDO2 inhibitors as shown in Table 2. In addition to being one of the best scoring hits on both enzymes with best interaction with main reported amino acids in both pockets, Pitavastatin has the safest potential side effects and is commercially available. Moreover, Xu et al.^25^ has reported its anticancer effect via downregulating AKT and ERK signals. Accordingly, it was elected as the drug of choice for further in vitro evaluation in this study.

### Molecular Dynamics simulations

**Figure 5:**
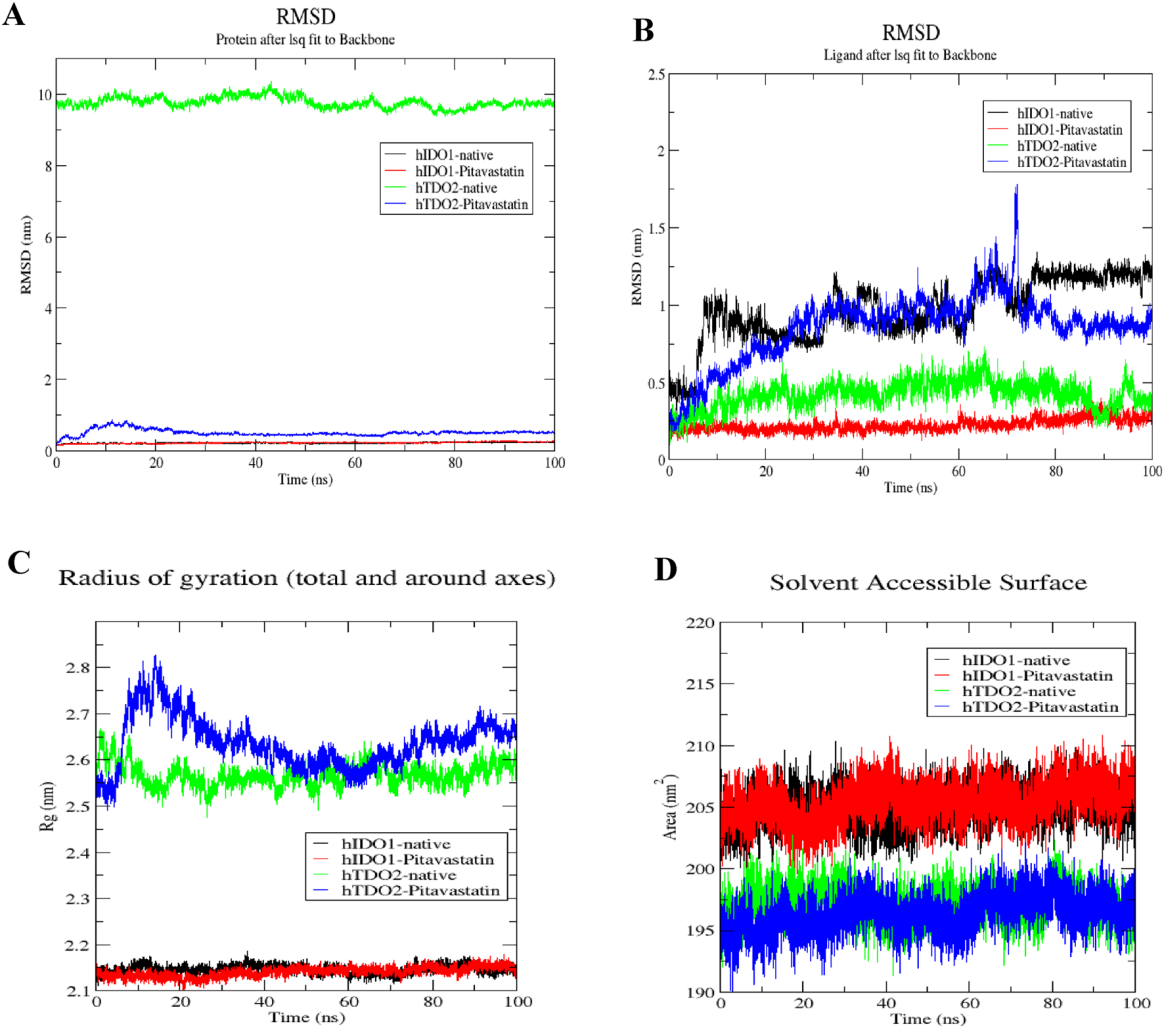
Structural dynamics of hIDO1 and hTDO2 RMSD upon binding to Pitavastatin and the co-crystalized ligands (**A**), ligands (Pitavastatin and the co-crystalized ligands) RMSD (**B**) hIDO1 and hTDO2 radius of gyration (**C**), SASA values (**D**) calculated during the 100 ns of MD trajectories.

To evaluate the stability of Pitavastatin binding to both target enzymes’ simulated system, we conducted a thorough examination of conformational changes within the protein–ligand complexes using three distinct methodologies: root-mean-square deviation (RMSD), radius of gyration (Rg), and solvent accessible surface area (SASA) analyses for both the ligand and the target throughout the 100 ns of molecular dynamics (MD) simulation (Fig. 5). These parameters were computed subsequent to re-centering and re-wrapping the complexes within the unit cells using the trjconv function within GROMACS.

The RMSD plot, shown in Fig. 5A, provides insight into the stability of the hIDO1 backbone in the presence of both Pitavastatin and its co-crystallized ligand (1-(4-cyanophenyl)-3-[[3-(2-cyclopropylethynyl)imidazo[2,1-b][1,3]thiazol-5-yl]methyl]urea), elucidating conformational changes over the course of the 100 ns MD simulation. Notably, the fluctuation pattern of the hIDO1 backbone in both scenarios closely aligns, demonstrating minimal fluctuation observed for both (depicted in black and red). Similarly, the backbone RMSD of hTDO2 was examined in complex with Pitavastatin and its co-crystallized ligand ((3S)-3-(5-fluoro-1H-indol-3-yl)pyrrolidine-2,5-dione). Binding of Pitavastatin (depicted in blue) resulted in enhanced stability compared to the co-crystallized ligand (depicted in green), with a slightly higher fluctuation observed.

To further validate their relative stability, the ligand RMSD plots upon binding to their respective target receptors (hIDO1 and hTDO2) were generated as referred in Fig. 5B. Remarkably, a notably higher stability was observed upon Pitavastatin binding to the hIDO1 receptor (depicted in red), exhibiting minimal fluctuation compared to the native ligand (depicted in black). For hTDO2, Pitavastatin demonstrated binding to the pocket with relatively high stability approximately after 20 ns from the simulation onset (depicted in blue), oscillating around only 0.1 nm, indicating a very stable interaction.

The compactness of the protein backbone within the four complexes was assessed by visualizing their radius of gyration (Rg) over the entire simulation duration (Fig. 5C). Consistently stable protein backbone compactness was observed for both Pitavastatin and the native ligand upon binding to the hIDO1 receptor (depicted in red and black, respectively). The protein compactness pattern for hTDO2 backbone mirrored the trends observed in the RMSD plot, with high backbone compactness observed following Pitavastatin binding to hTDO2, particularly evident after 20 ns. This stability was further corroborated by SASA plots, demonstrating minimal fluctuation ranging between 200 and 210 nm^2^ for the two hIDO1 complexes and between 190 and 200 nm^2^ for hTDO2 complexes throughout the entire simulation duration (Fig. 5D).

**Figure 6:**
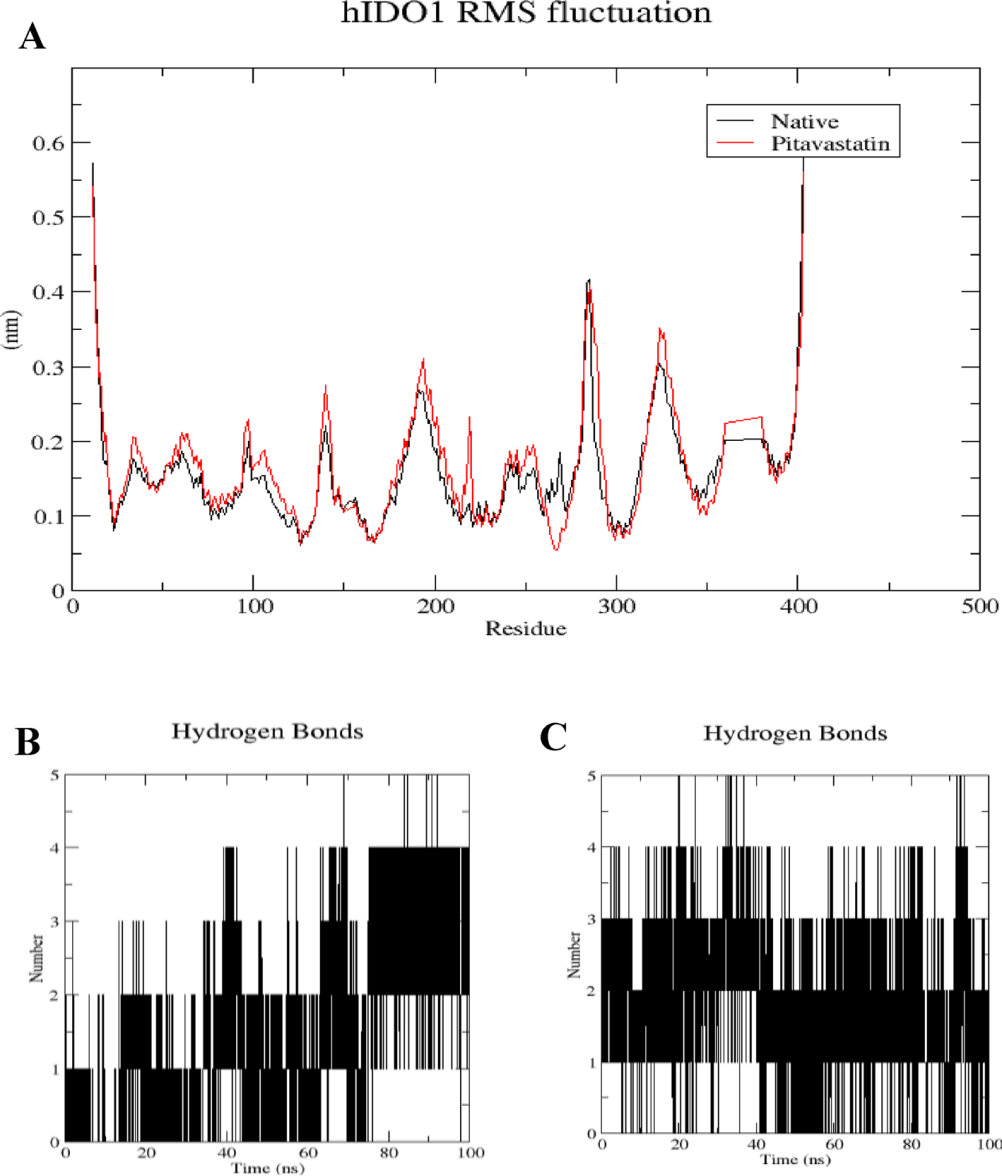
Structural dynamics calculated during the 100 ns of MD trajectories; Root Mean Square fluctuation (RMSF) of hIDO1 protein backbone (**A**), number of H-bonds formed with **hIDO1 native ligand** (**B**) and with **Pitavastatin** (**C**).

**Figure 7:**
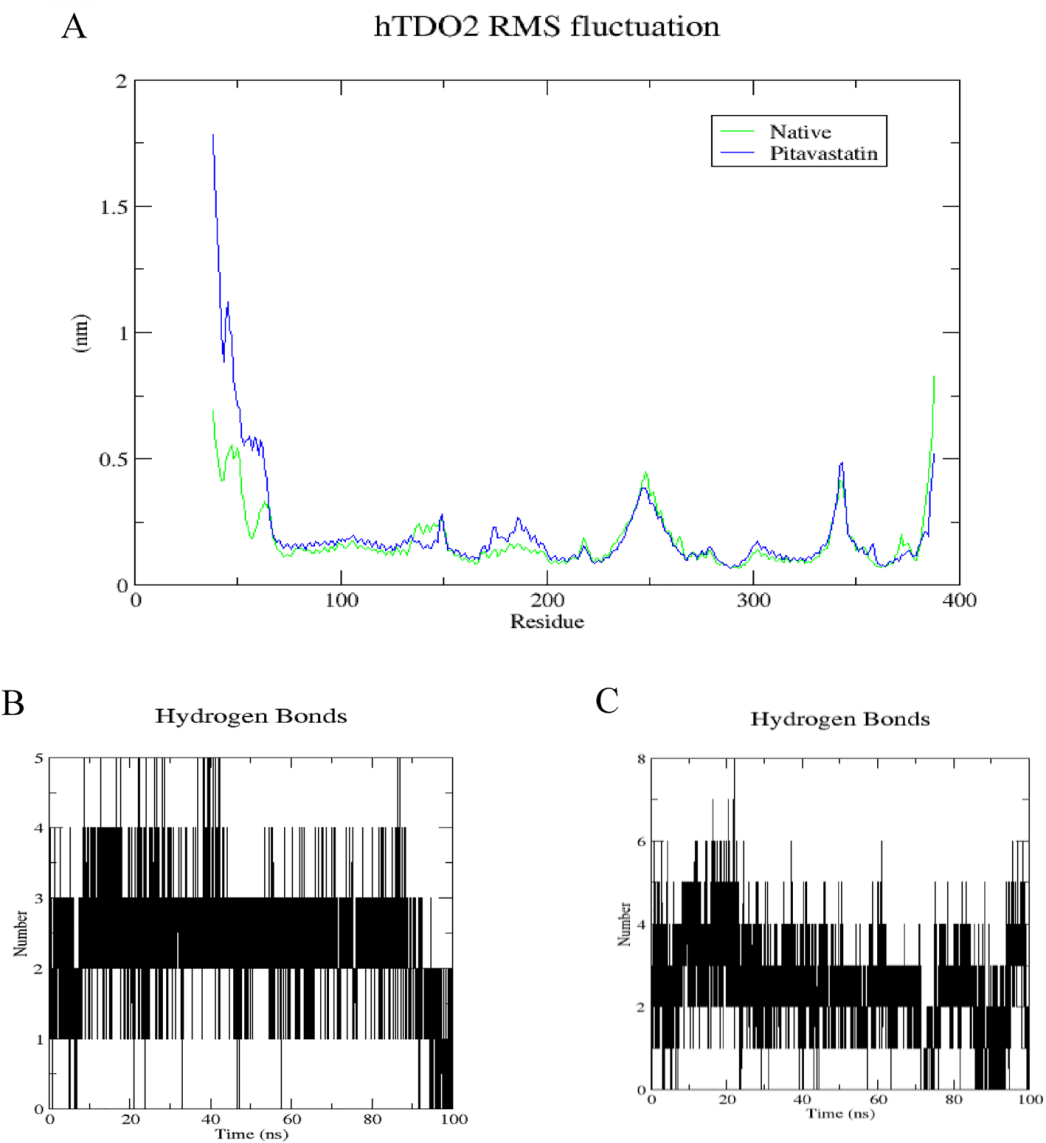
Structural dynamics calculated during the 100 ns of MD trajectories; Root Mean Square fluctuation (RMSF) of hTDO2 protein backbone (**A**), number of H-bonds formed with **hTDO2 native ligand** (**B**) and with **Pitavastatin** (**C**).

The root mean square fluctuation (RMSF) analysis was conducted to assess the rigidity and flexibility of backbone residues within hIDO1 and hTDO2 throughout the 100 ns of molecular dynamics (MD) simulation. Figures 6A and 7A depict the RMSF patterns for the two hIDO1 complexes and the two hTDO2 complexes, revealing consistent trends across both proteins. Notably, residues involved in ligand interactions exhibited minimal fluctuation, typically less than 0.2 nm, indicative of stable binding interactions. For hIDO1, the two key residues with minimal fluctuations are Gly262 (approximately 0.05 nm) and Arg231 (around 0.1 nm). For hTDO2, the key residues with small fluctuations are His76 (less than 0.2 nm), Arg144(less than 0.15 nm), and Thr342 (around 0.2 nm). Overall, both results are highly consistent with the docking study results illustrated in Fig. 4.

To further elucidate the stability of these interactions, the number of hydrogen bonds formed during the 100 ns simulation was quantified (refer to Figs. 6B, C, 7B, and C). In the case of hIDO1, the co-crystallized ligand maintained 1–2 hydrogen bonds during the initial 75 ns of the simulation, whereas Pitavastatin demonstrated a larger number of stable hydrogen bonds (2–3) throughout the entire simulation, with occasional formation of a third and fourth bond in certain frames. Similarly, for hTDO2, an average of 2–4 hydrogen bonds was observed throughout the trajectory, correlating with the high stability observed in both RMSD and Rg plots. Pitavastatin also exhibited the ability to form stable 2–4 hydrogen bonds over the entire 100 ns simulation duration.

These findings underscore the favorable binding affinity of Pitavastatin and the co-crystallized ligands to their respective target receptors, highlighting the importance of stable hydrogen bond formation in driving robust ligand-receptor interactions.

### In vitro hIDO1 and hTDO Inhibitor Screening Assay

**Figure 8:**
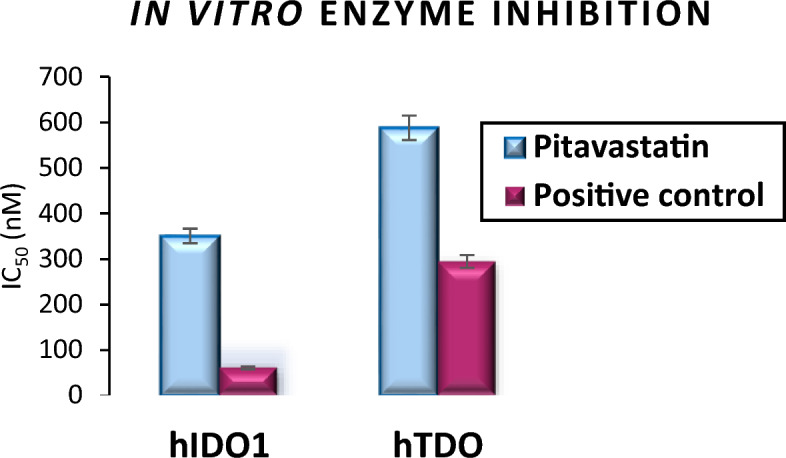
A graphical representation for Pitavastatin inhibition activity against hIDO1 and hTDO2 enzymes compared to the positive control, represented in IC_50_ (nM).

Two in vitro enzymatic inhibition assays were selected to assess Pitavastatin inhibition activity against hIDO1 and hTDO2 enzymes and compare this inhibition activity to that of the positive control. IC_50_ value*s* listed in Table 4 and Fig. 8 confirm the previous findings of the docking study where Pitavastatin showed an inhibition activity against both enzymes in nM (351 and 588 nM for hIDO1 and hTDO2, respectively) confirming its dual inhibition activity being more potent towards hIDO1.

**Table 4:** IC_50_ of Pitavastatin Calcium and the positive control against hIDO1 and hTDO2.

Compound	hIDO1 (nM)	hTDO2 (nM)
Pitavastatin-Ca	351 ± 16	588 ± 27
Positive control	61 ± 3	295 ± 14

### MTT cytotoxicity assay

**Figure 9:**
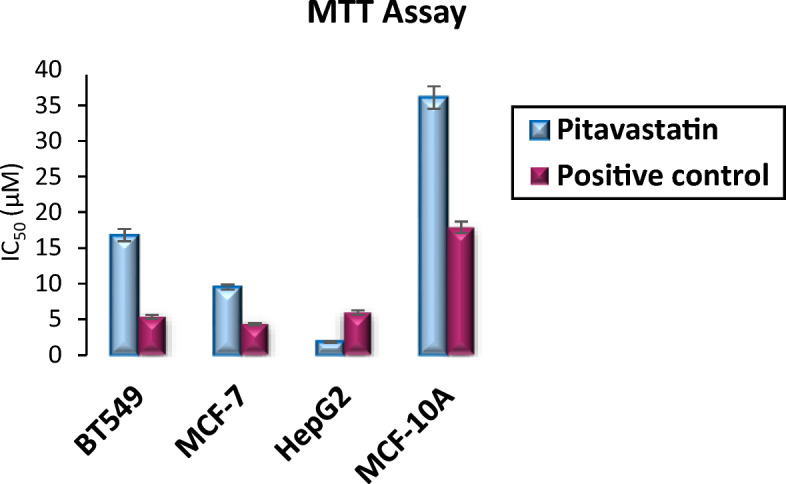
A graphical representation of cellular *IC*_*50*_ of Pitavastatin compared to control against BT-549, MCF-7, HepG2 and MCF-10A cell lines.

As shown in Table 5 and Fig. 9, Pitavastatin demonstrated a potent cytotoxic effect on all three tested cancer cell lines BT-549, MCF-7, and HepG2, respectively. However, the highest response was that of the liver cancer HepG2 cell lines which were almost 9.14-fold more sensitive than BT-549 and 5.17-fold more responsive than MCF-7 to Pitavastatin treatment.

**Table 5:** IC_50_ values of Pitavastatin on BT-549, MCF-7, HepG2 and MCF-10A cells.

Sample	Cytotoxicity (IC_50_ ± SD) (µM)			
	BT-549	MCF-7	HepG2	MCF-10A
Pitavastatin-Ca	16.82 ± 0.86	9.52 ± 0.35	1.84 ± 0.1	36.07 ± 1.56
Staurosporine (Positive control)	5.35 ± 0.27	4.32 ± 0.16	5.92 ± 0.32	17.92 ± 0.78

Additionally, Pitavastatin showed to be highly selective affecting higher cytotoxicity to cancer cell lines BT-549, MCF-7, and HepG2 in contrast to its lower toxic effect on the normal cell line MCF-10A with IC_50_ values of 16.82, 9.52, 1.84, and 36.07 µM, respectively. Pitavastatin is 2.14-fold selective to BT-549 over MCF-10A, 3.79-fold selective to MCF-7, and 19.6-fold selective to HepG2 cells. Conclusively, Pitavastatin demonstrated a high safety margin and selective cytotoxicity towards different cancer cell lines with little cytotoxicity towards the tested normal cell line.

The mechanism underlying Pitavastatin's dual inhibitory action on hIDO1/hTDO2 is of particular interest. By targeting multiple nodes within Trp catabolic pathway, Pitavastatin may offer a more effective and comprehensive approach to disease management. hIDO1 and hTDO2 play key roles in tryptophan metabolism, which is implicated in immune regulation and tumor immune escape. By inhibiting both enzymes, Pitavastatin could disrupt Trp metabolism in cancer cells, potentially altering the tumor microenvironment and enhancing immune surveillance against cancer cells^26^. This dual inhibition may contribute to Pitavastatin's anticancer activity observed across different types of cancer.

Statins have been reported to have an anticancer effect due to their antioxidant, anti-inflammatory and antiproliferative activities against various types of cancer cells like ovarian, breast, prostate, colon, lung, brain, cancers, and melanoma^27^. They were also found to have a synergetic effect when combined with other chemotherapies like doxorubicin, topotecan and paclitaxel^28^. In addition to their anticancer effect, patients using statins reported low risks for cancer development as high cholesterol levels are correlated with cancer development^29^. Furthermore, Pun et al.^30^ reported the apoptotic activity (autophagy-mediated) of Pitavastatin upon treatment in oral squamous cell carcinoma cells (SCC15) and colon cancer cells (SW480).

To contextualize its efficacy and compare it to existing inhibitors, we can examine previous studies and clinical trials on both single-target and dual inhibitors of hIDO1 and hTDO2. Single-target inhibitors of hIDO1 and hTDO2, such as epacadostat (hIDO1 inhibitor) and navoximod (hTDO2 inhibitor), have been investigated extensively in preclinical and clinical settings. For example, epacadostat was evaluated in combination with immune checkpoint inhibitors in various solid tumors, including melanoma and lung cancer. Despite promising preclinical data suggesting synergy between hIDO1 inhibition and immune checkpoint blockade, clinical trials failed to demonstrate significant improvements in overall survival or progression-free survival compared to standard of care treatments^31–33^. Similarly, navoximod showed limited efficacy as a single agent in early-phase clinical trials, prompting further exploration of combination strategies^32^.

In contrast, dual inhibitors of hIDO1 and hTDO2 offer a unique advantage by targeting two key enzymes involved in tryptophan metabolism within the tumor microenvironment. Preclinical studies have shown that simultaneous inhibition of hIDO1 and hTDO2 may lead to more pronounced anticancer effects compared to single-target inhibitors. For example, a recent study by Li et al.^34^ demonstrated that dual inhibition of hIDO1 and hTDO2 using a small molecule inhibitor resulted in enhanced antitumor immune responses and tumor regression in mouse models of melanoma and colon cancer. This suggests that targeting both enzymes simultaneously may overcome potential compensatory mechanisms and improve therapeutic outcomes.

### Flow cytometry apoptosis assay and cell cycle analysis

Since MTT cytotoxicity assay unveiled the significant cytotoxic effect of Pitavastatin on the three tested cancer cell lines, further evaluation was needed to investigate the mechanism of cell death. HepG2 was selected for all further in vitro mechanistic investigations as it was the most affected cancer cell line when treated with Pitavastatin (showing very low IC_50_ value = 1.84 µM).

**Figure 10:**
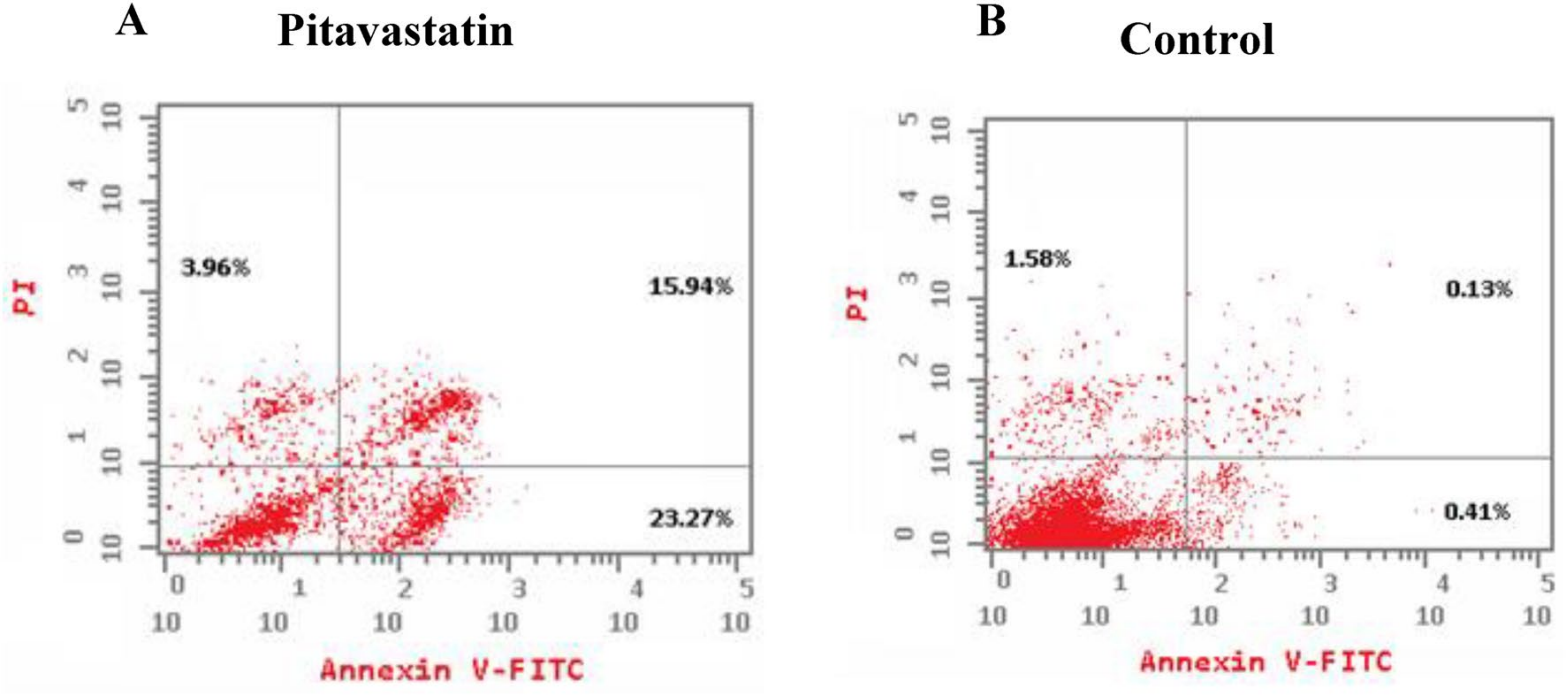
Induction of apoptosis in HepG2 cells by annexin-V/propidium iodide stain. Cytograms showing HepG2 cells treated with Pitavastatin (1.84 µM, 48 h) (**A**), HepG2 cells as an untreated control (**B**).

Apoptotic and necrotic cell population percentage was determined for HepG2 cell line after 48 h of treatment with the pre-calculated IC_50_ values of Pitavastatin via annexin-FITC/propidium iodide (AV/PI). Cell apoptosis assay results on HepG2 cells (Fig. 10) demonstrate the percentage of live cells decreased from 97.88% (control) to 56.83% when treated with Pitavastatin. Total cell death is reported to be 43.17%, whereas the sub-populating in early apoptosis increased to 23.27%, the average late apoptotic sub-population count reached 15.94% and an increase in necrosis was also observed (about 2.5 times more than control untreated cells). The results proved that the Pitavastatin effectively induced apoptosis, substantially early apoptosis followed by late apoptosis, in HepG2 cells. Similar results were obtained by Abdullah et al.^35^ reporting significant early or late apoptosis when Pitavastatin was applied in combination with Prednisolone on ovarian cancer cell lines (Ovcar-4 and Cov-362). Likely, late apoptotic/necrotic phase cell death was observed as a result of the synergetic combination of Pitavastatin and Gemcitabine on human MIA PaCa-2 cell line (pancreatic carcinoma)^36^. Also, late apoptosis was observed upon incubating preactivated T-cells with high concentration of Pitavastatin as a result of caspase-3/7 activation^37^.

**Figure 11:**
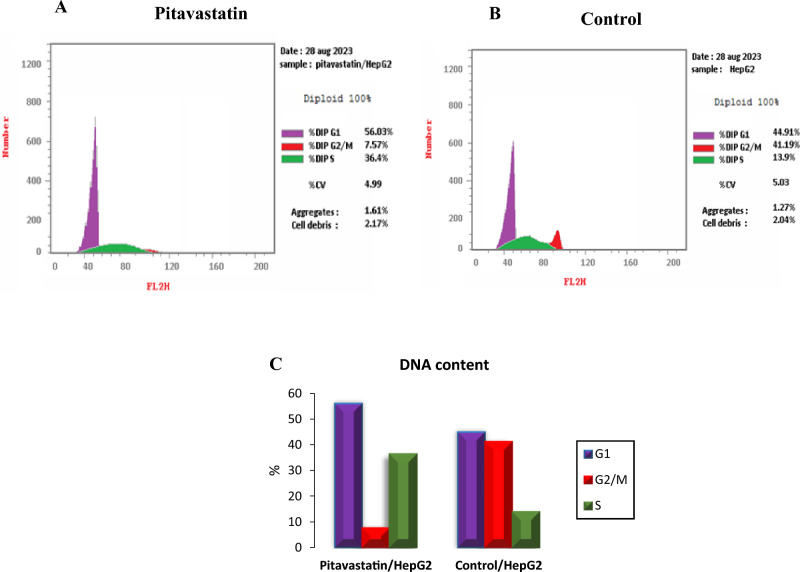
Cell cycle analysis cytograms of Pitavastatin-treated HepG2 cells (**A**), control untreated HepG2 cells (**B**) and percentage of HepG2 cell population in various cell cycle phases (**C**).

Afterwards, a cell cycle analysis test was utilized to study the cell proliferation percentage in different cell cycle phases. The highest cell arrest induction was observed in G1 and S phases with 1.11-fold and 1.14-fold, respectively (52.61% and 44.01%, respectively) when compared to the control as shown in Fig. 11. These results affirm that Pitavastatin induces G1/S phase arrest in HepG2 cancer cell line. Pitavastatin was also reported to induce S-phase cell cycle arrest in human U937 monocytic tumor cells^38^. Additionally, cell apoptosis via G1 cell cycle arrest was noticed during combined Pitavastatin- dacarbazine treatment^39^.

### ELISA assay and RT-PCR for cell cycle regulators

**Figure 12:**
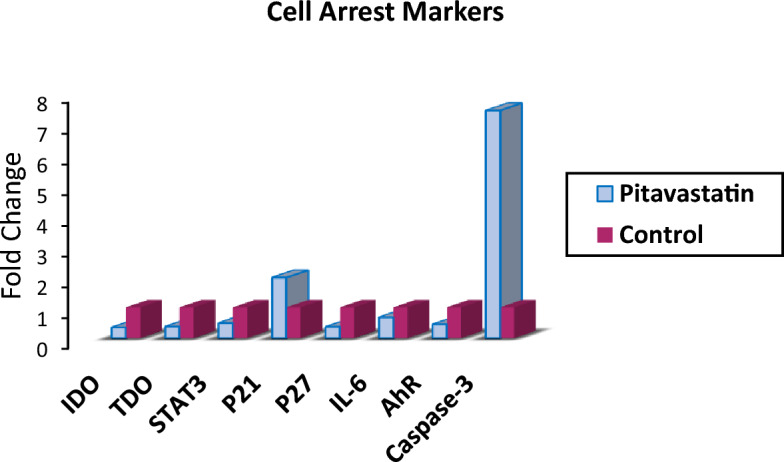
A graphical representation of different Cell arrest markers expression upon Pitavastatin treatment on HepG2 cells.

The expression levels of hIDO1, hTDO2, STAT3, P21, P27, IL-6, AhR and Caspase3 as cell cycle regulators and markers of cell cycle arrest were measured using ELISA Kit to demonstrate the mechanism by which Pitavastatin inhibits HepG2 cells survival. As shown in Table 6 and Fig. 12, treatment with Pitavastatin has affected significant downregulation of the expression of both hIDO1 and hTDO2 by 2.9- and 2.7-fold, respectively, thus, affirming the dual hIDO1/hTDO2 inhibition capability of Pitavastatin.

**Table 6:** Cell cycle regulators expression upon Pitavastatin treatment on HepG2 cells.

Assay	ELISA (pg/ml)						RT-PCR (Fold change)	
Cell cycle regulators	IDO	TDO	STAT3	P21	P27	IL-6	AhR	Casp3
Pitavastatin	605.3 ± 20.8	755 ± 3	5850 ± 26	406.7 ± 15.7	2.923 ± 0.24	91.83 ± 1.99	0.447	7.4035
Control	1754 ± 26.3	2032 ± 5	11,880 ± 170	206.5 ± 8.41	8.07 ± 1.3	135.3 ± 2.93	1	1

Previous studies reported that high hIDO1 levels in various cancer cells are induced by IL-6 and others reported that hIDO1 can activate and upregulate IL-6 at the early stage of tumorigenesis to influence inflammatory conditions and facilitate cancer growth^40^. Also, the inhibition of IL-6 using siRNA and/or pharmacological inhibitors was found to reduce IDO mRNA and protein expression as well as kynurenine formation^41^. Accordingly, IL-6 expression is a critical biomarker to evaluate and confirm the anticancer capability of Pitavastatin against HepG2. Indeed, treating HepG2 cells with the predetermined IC_50_ value has resulted in lowering the level of IL-6 (91.83 pg/mL) compared to control untreated cells (135.3 pg/mL).

The effects of the IDO-AHR-IL-6-STAT3 loop in HepG2 were also evaluated. Previous research suggests that targeting the IDO-AHR-IL-6-STAT3 loop may reverse immune suppression caused by IDO1^42–45^. Sustained hIDO1 expression requires constitutively active STAT3, which is regulated by autocrine IL-6 through phosphorylation and acetylation^46^. Our findings indicate a decrease of approximately 50% in STAT3 and 55% in AhR, confirming Pitavastatin’s effective therapeutic intervention with the immune suppression loop. AhR downregulation is essential, as activation of AhR may result in pro-cancerous effects in several human malignancies and may have a role in poor prognosis.^47,48^. Additionally, some studies have shown that cancer cells that overexpress hTDO2 and activate AhR are able to evade immune surveillance^49^.

The pro-apoptotic effect of inhibiting hIDO1 and hTDO2 is correlated with p21 and p27 inhibition. hIDO1/AhR pathway deactivation was found to downregulate the cell cycle inhibitor p27 which induces G1/S phase blockage^50,51^. In our study a 63.78% reduction in p27 expression was observed for Pitavastatin treated HepG2 cells compared to the control untreated cells as shown in Fig. 12. On the other hand, p21 expression increased 2 times more than the control, however, hIDO1 inhibition was previously reported to decrease p21 expression in T cells which in turn leads to apoptosis^52^. This abnormal overexpression of p21 in HepG2 after Pitavastatin treatment was also previously reported in human U937 monocytic tumor cells treated with Pitavastatin^38,39^. It remains unknown whether this high p21 level affects cancer progression so further investigation is needed.

Elevated levels of caspase-3 are widely recognized to induce cellular apoptosis by cleaving various downstream substrates, resulting in characteristic morphological changes that serve as a significant marker of apoptosis^53^. In this study, caspase-3 level increase 7.4-fold upon Pitavastatin treatment compared to the control untreated cells.

## Conclusion

In this forward-looking study, a meticulously crafted hIDO1/hTDO2 ligand-based pharmacophore model was designed and achieved a notable accuracy rate of 89.73%. This model was put to the test against a vast library of 2658 FDA-approved drugs, identifying 308 compounds that not only satisfied the stringent criteria of the pharmacophore search but also adhered to Lipinski's and Veber's rules for oral bioavailability. Through intensive virtual screening against the crystal structures of hIDO1 and hTDO2, the pinnacle of four top-scoring ligands emerged: Trovafloxacin, Vilazodone, Pitavastatin, and Dasabuvir. Pitavastatin, by virtue of its favorable binding energy score and substantial interactions with critical residues in both enzymes along with its tolerable safety profile, emerged as the optimal lead compound. Our subsequent molecular dynamics simulations offered profound insights into the stability of Pitavastatin's binding to hIDO1 and hTDO2 receptors, unveiling the intricate dynamics of drug-protein interactions. Rigorous analyses, including root-mean-square deviation (RMSD), radius of gyration (Rg), and solvent accessible surface area (SASA), consistently indicated stable binding events, affirming Pitavastatin's potential.

Demonstrating potent cytotoxicity against BT-549, MCF-7, and HepG2 cell lines while exhibiting a remarkable safety margin on normal breast cells (MCF10-A), Pitavastatin emerged as a formidable contender in cancer therapy being most potent against HepG2 cells. Its anticancer prowess was observed to disrupt the G1/S phase, inducing apoptotic pathways through caspase-3 activation, and orchestrating the downregulation of pivotal cellular regulators such as STAT3, P21, P27, IL-6, and AhR, thereby impeding tumor progression. This multifaceted approach extended to bolstering anti-tumor immunity by tempering the immunosuppressive interleukin IL-6. In conclusion, Pitavastatin emerges as a compelling candidate for anticancer therapy, targeting both hIDO1 and hTDO2 enzymes, thus unveiling a novel mechanism of action. While computational advancements continue to expand the horizons of drug discovery, the indispensable need for experimental validation of compound activity and safety underscores the importance of meticulously paced research. The fusion of pharmacophore modeling and virtual screening in drug repurposing endeavors serves as a guiding light in the pursuit of innovative therapies, poised to revolutionize the medical treatment landscape.

### Future perspectives

Computational-aided drug design (CADD) methodologies have been effectively crafted to enhance the capacity for uncovering novel pharmaceuticals. Advancements in computational techniques are broadening the scope of potential applications, facilitated by the increased accessibility of protein target structures via deep learning and AI-driven prediction tools^54^. Recent strides have seen the integration of deep learning techniques into the exploration of drug-target interactions^55^. From this vantage point, the strategy of pharmacophore modeling followed by virtual screening of approved drug databases, particularly within the framework of drug repurposing, emerges as the forefront rational approach. This method holds the most promise in uncovering novel therapies, offering a streamlined pathway amidst the complex landscape of drug development.

### Supplementary Information

Supplementary Information.

## Data Availability

The datasets used and/or analyzed during the current study will be available from the corresponding author upon reasonable request.
