# Supplementary material for: Exploring a repurposed candidate with dual hIDO1/hTDO2 inhibitory potential for anticancer efficacy identified through pharmacophore-based virtual screening and in vitro evaluation
Source: Sci Rep. 2024 Apr 24;14:9386. doi: 10.1038/s41598-024-59353-4 (PMC11039737; doi:10.1038/s41598-024-59353-4)
Supplement: Supplementary file 1 — Supplementary Information. [file 41598_2024_59353_MOESM1_ESM.docx]

**Supplementary Materials**

**Exploring a Repurposed Candidate with Dual hIDO1/hTDO2 Inhibitory Potential for Anticancer Efficacy Identified through Pharmacophore-Based Virtual Screening and *In Vitro* Evaluation**

**Nourhan M. Aboomar ^a,b,#^, Omar Essam ^a,b,#^, Afnan Hassan ^a,b,c#^, Ahmad R. Bassiouny ^d^, Reem K. Arafa ^a,b,*^**

^a^ Drug Design and Discovery Lab, Zewail City of Science and Technology, Cairo, 12578, Egypt

^b^ Biomedical Sciences Program, University of Science and Technology, Zewail City of Science and Technology, Cairo, 12578, Egypt

^c^ Euro-Mediterranean Master in Neuroscience and Biotechnology Program, Alexandria University, Alexandria, 21511, Egypt

^d^ Department of Biochemistry, Faculty of Science, Alexandria University, Alexandria, 21511, Egypt

- 1. **High throughput virtual screening**

For hIDO1, according to the ligand-protein interactions demonstrated in **Figure S1**, the most two essential residues to be inhibited are Arg231 and Ser167. Five hits out of ten are shown to create H-bond acceptor or hydrophobic interactions with Arg231, while H-bond donor or acceptor interactions with Ser167 are present in 2 drugs out of 10. In addition, H-bond donor interaction with Cys129 is another main interaction found in 2 hits out of 10. Also, two drugs out of ten formed H-bond acceptor or hydrophobic main interactions with Tyr126. The π-π interaction with Phe163 and H-bond donor with Gly262 backbone are the other two main interactions found in one hit and two hits out of ten, respectively. Furthermore, three out of ten were detected to interact with the Lys238 backbone as a H-bond/acceptor. In addition, H-bond donor/acceptors and H-arene interactions with ser235 were visualized in 3 hits of 10. Also, H-arene and H-bond acceptor interactions with Gly236 were detected in 3 hits out of 10. Five drugs out of ten interacted with the heme group via H-bond, π-π, or π-H interactions. This highly aligns with the previous studies demonstrating the Ser167 essential role as a catalyst for L-Trp catabolism and the importance of Arg231 in L-Trp binding recognition. Also, the presence of H-bond donor interaction with Gly262, H-bond acceptor with Tyr126, and π-π interaction with Phe163 in the hits increase the drug affinity to the receptor and their inhibition capability [1].

All four hits form two main interactions with the active site residues as a minimum. Pitavastatin forms two essential interactions. Likewise, dasabuvir creates two main interactions with additional three hydrophobic and π-π interaction with the heme group. Trovafloxacin forms three interactions with another H-bond with heme. Finally, Vilazodone has six interactions; four of them are main interactions. Ranolazine formed only one H-bond donor interaction while Acetophenazine formed an H-bond acceptor and an H-Arene interaction, and Dasatinib has an H-bond acceptor interaction with a non-essential residue (**Figures 1E, 1F, and 1G).** Also, Gefitinib creates two H-Arene interactions with two residues while Apixaban forms one H-bond acceptor interactions with an essential Amino acid (**Figures 1H and 1I)**. Finally, Nefazodone forms two H-Arene interactions and a H-bond acceptor with three non-essential residues, as demonstrated in **Figure 1J.**


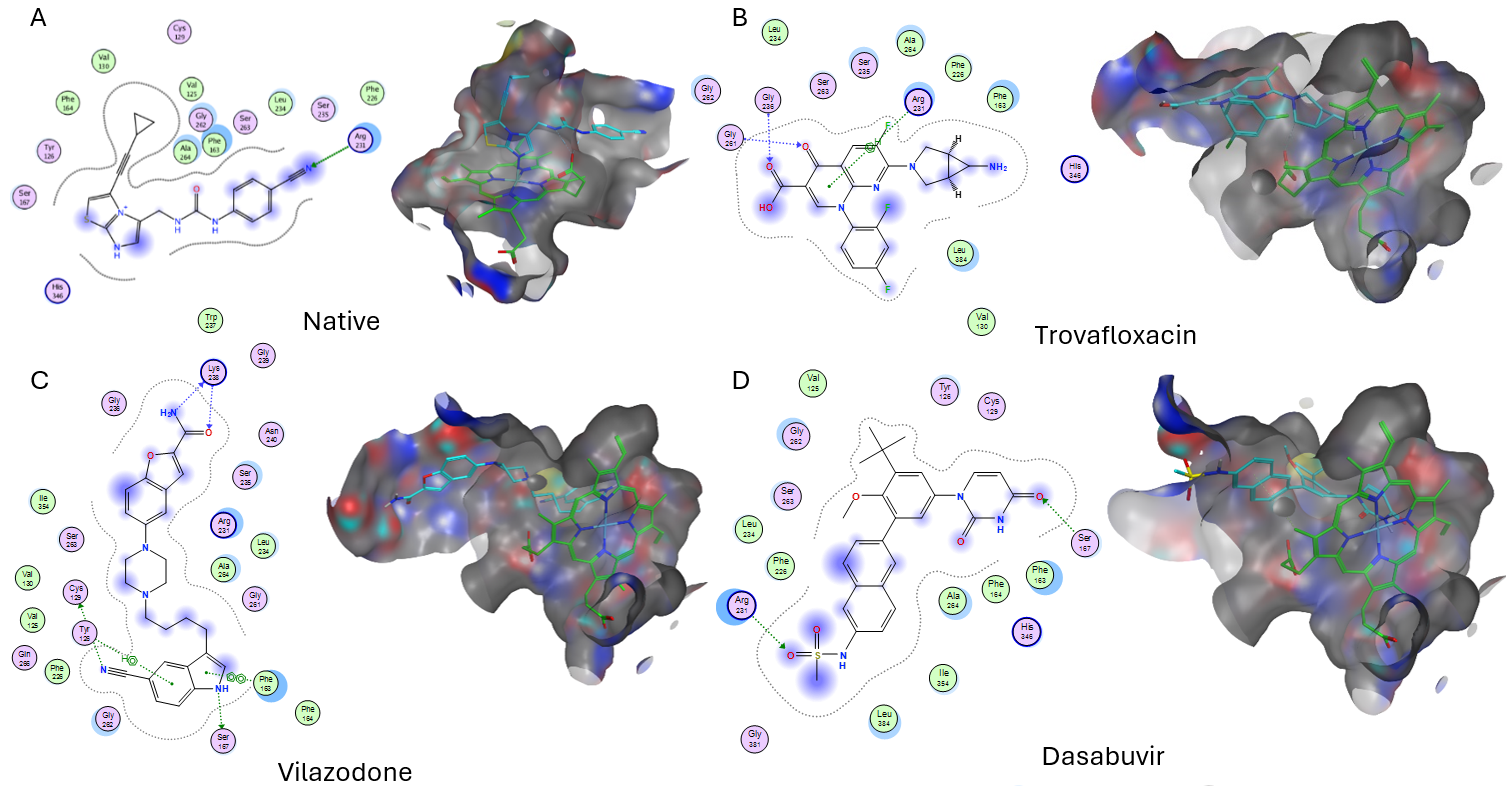

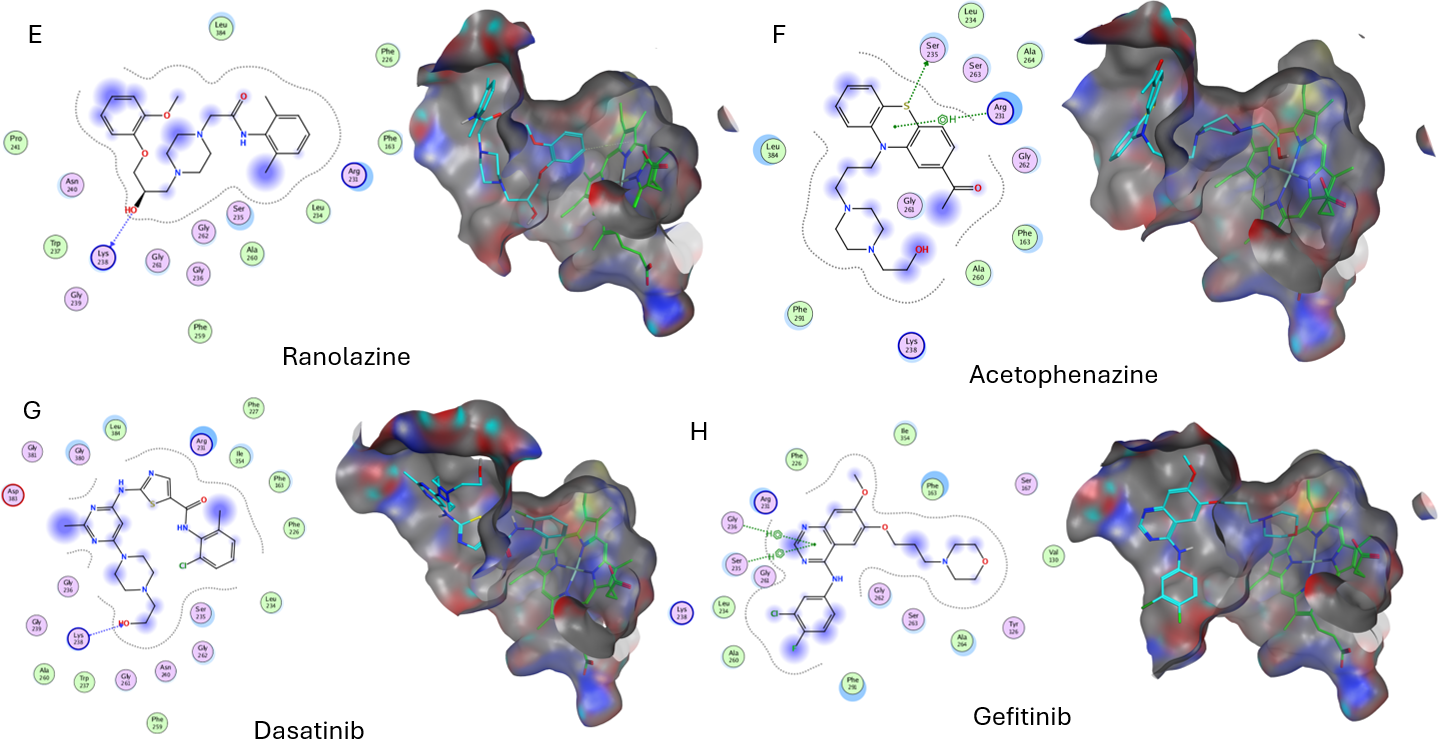

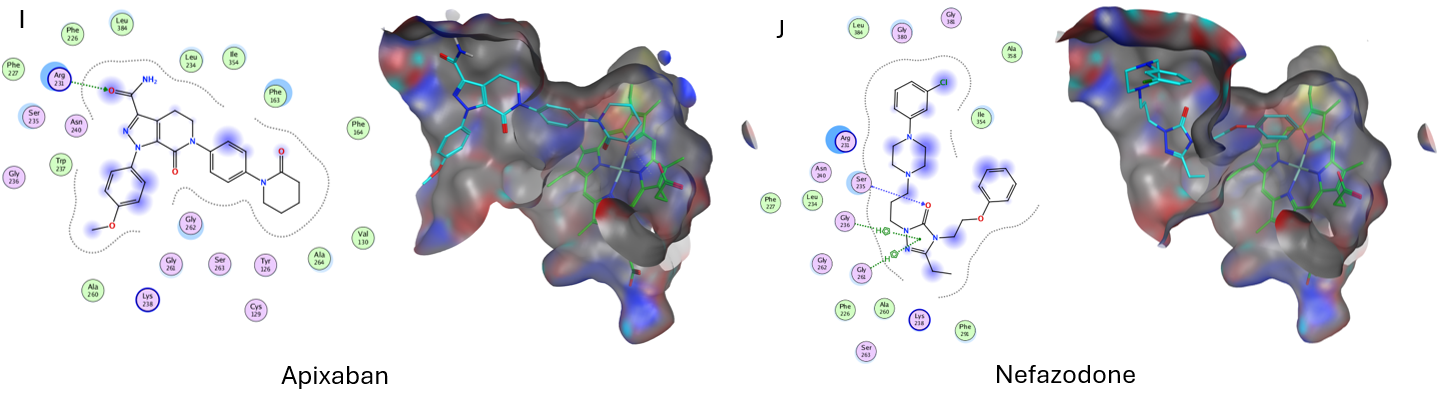


**Figure S1:** 2D interactions and pocket surface occupancy of the native ligand (A) and some of the top hits docked from the filtered Drug data bank database against hIDO1 (B, C, and D). (Heme is shown as green, the hits are shown as Cyan, the surface map is the Pocket surface occupancy, in which the blue color represents cationic moieties, while red color represents anionic moieties. The hydrophobic moieties are illustrated in gray).

For hTDO2, based on the ligand-receptor interaction results illustrated in **Figure 5**, H-bond acceptor interaction with Arg144 is essential due to its presence in all top four hits out of ten. Also, the π-π or π-H interactions with His76 and in the pocket are essential and found in four hits out of ten. The results are consistent with the previous studies reporting the His76 crucial role as a base catalyst responsible for L-Trp catabolism, in addition to Arg144's importance in L-Trp recognition [2]. Moreover, H-bond acceptor interaction with the N-terminal of Thr342 and H-bond donor interaction with its OH group are other main interactions being observed in four drugs out of ten. Three of them were H-bond acceptors with Thr342 backbone. Another possible main interaction detected in two drugs out of ten is the H-bond donor with the Glu80 sidechain. Moreover, an H-bond/ acceptor interacting with the C-terminus of Glu56 was confirmed in two drugs out of ten. In addition, the H-arene or a H-bond acceptor interactions with Ser151 sidechain and N-terminus, respectively, were visualized in three hits out of ten while an H-arene interactions with Ile73 sidechain was found in two drugs out of ten. Also, three out of ten were detected to form an H-bond acceptor interaction with the N-terminus of Gly152. Eventually, six hits out of ten could form at least one main hydrophobic interaction with the heme group, which is essential for hTDO2 activity.

All top ten FDA-approved drugs form at least one interaction; four of them create at least two essential interactions with the pocket amino acids besides an additional main interaction with the heme group. Pitavastatin can create 4 interactions with three amino acids; all of them are the main interactions (**Figure 4B in MS**). Vilazodone binds to the pocket residues via four main interactions, while Dasabuvir forms three main ones **(Figures 2C and 2D)**. Also, Trovafloxacin creates two main interactions, as shown in **Figure 2B**. On the other hand, the other six hits could not form the essential interactions. Ranolazine formed only one H-bond donor interaction while Acetophenazine formed two H-bond acceptor interactions with non-essential residues, and Dasatinib has only two H-Arene interactions (**Figures 2E, 2F, and 2G).** Also, Gefitinib creates two interactions with two residues while Apixaban forms three interactions with two non-essential Amino acids (**Figures 2H and 2I)**. Finally, Nefazodone forms two H-Arene interactions with two residues, including His76, which is considered a critical amino acid, as illustrated in **Figure 2J.**


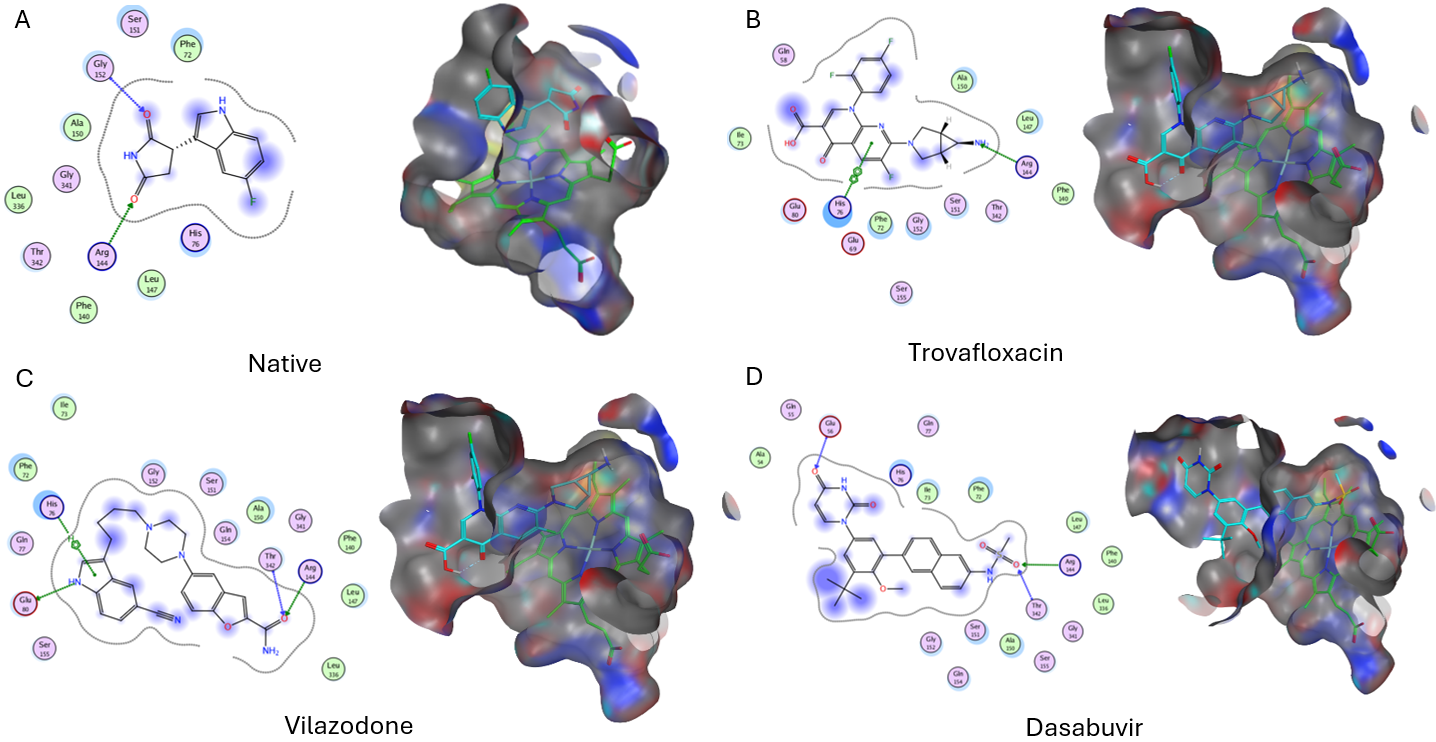

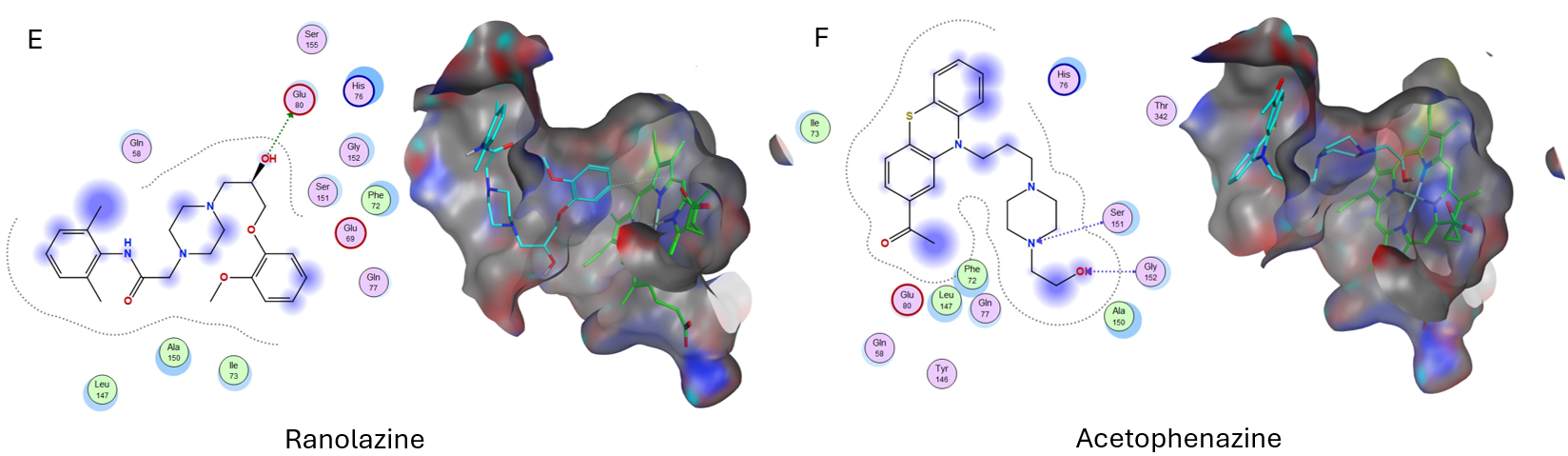

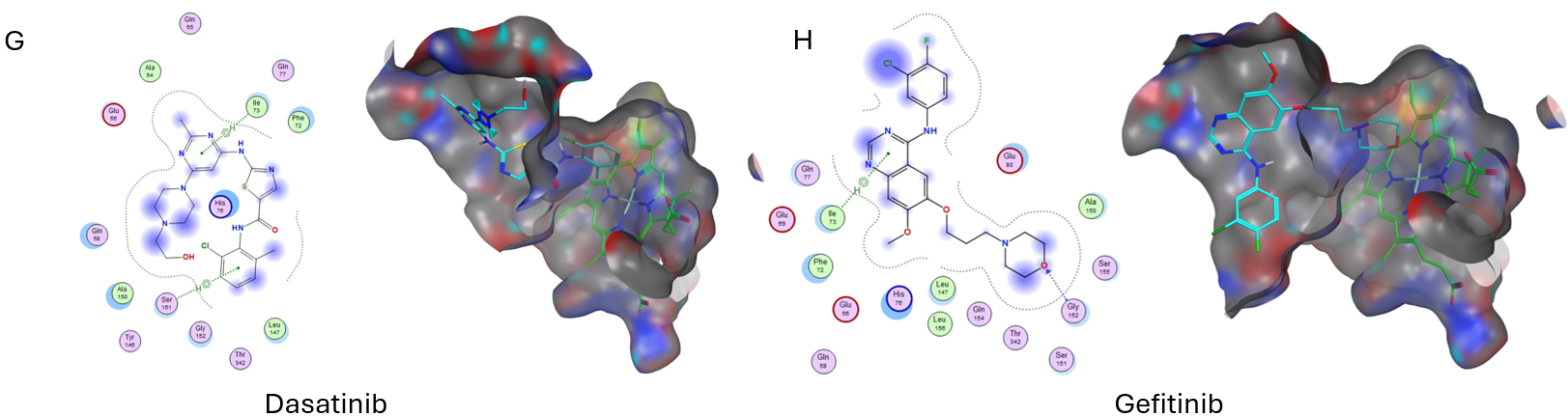


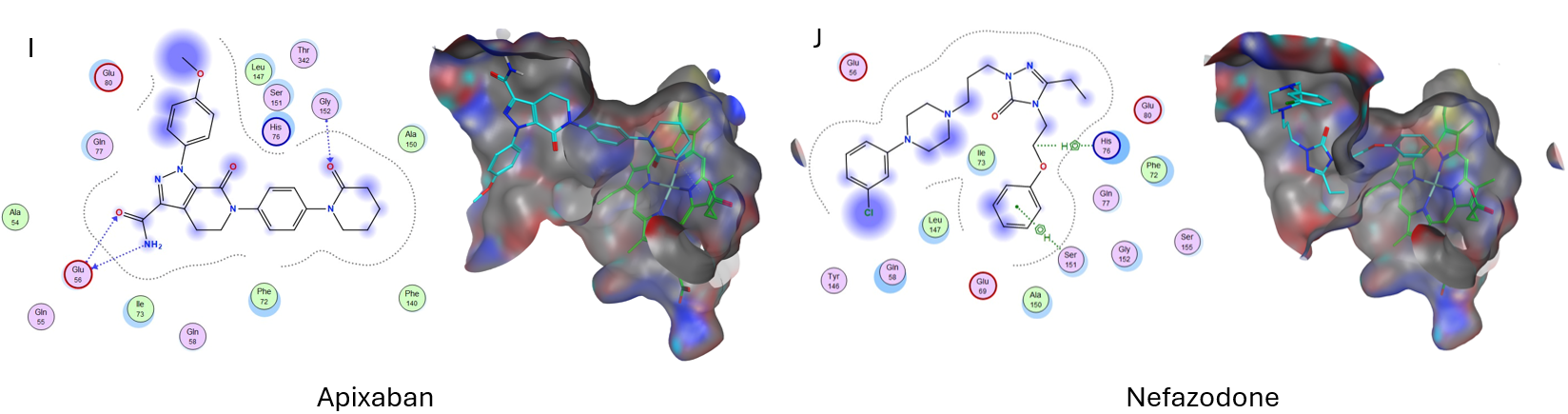


**Figure S2:** 2D interactions and pocket surface occupancy of the native ligand (A) and some of the top hits docked from the filtered Drug data bank database against hTDO2. (Heme is shown in green, the hits are shown in Cyan, the surface map is the Pocket surface occupancy, in which the blue color represents cationic moieties, the red color represents anionic moieties, and the hydrophobic moieties are illustrated in gray).

**References**

1. Meng, B., et al., *Structural and functional analyses of human tryptophan 2,3-dioxygenase.* Proteins, 2014. **82**(11): p. 3210-6.

2. Pham, K.N., A. Lewis-Ballester, and S.-R. Yeh, *Conformational plasticity in human Heme-Based dioxygenases.* Journal of the American Chemical Society, 2020. **143**(4): p. 1836-1845.
